# Professional socialization as a complex adaptive process: a systematic scoping review and integrative theory of professional identity formation

**DOI:** 10.1186/s12909-026-09894-1

**Published:** 2026-07-10

**Authors:** Suzanne Pei Lin Goh, Mikail Faris Akbar, Anton Zhi Hao Teo, Priyadarshini A, Jun Rey Leong, Annushkha Sinnathamby, Eng Koon Ong, Vengadasalam Murugam, Ying Yin Lim, Crystal Lim, Muhammad Amin Shaik, Lalit Kumar Radha Krishna

**Affiliations:** 1 KK Women’s and Children Hospital, 100 Bukit Timah Road, Singapore, 229899 Singapore; 2 Duke-NUS Medical School, 8 College Road, Singapore, 169857 Singapore; 3 Yong Loo Lin School of Medicine, National University of Singapore, NUHS Tower Block, Level 11, Block 1E, Kent Ridge Road, Singapore, 119228 Singapore; 4 Division of Supportive and Palliative Care, National Cancer Centre Singapore, 30 Hospital Boulevard, Singapore, 168583 Singapore; 5 Division of Cancer Education, National Cancer Centre Singapore, 30 Hospital Boulevard, Singapore, 168583 Singapore; 6 Khoo Teck Puat National University Children’s Medical Institute, National University Health System, 5 Lower Kent Ridge Road, Singapore, 119074 Singapore; 7 Division of Supportive and Palliative Care, National University Cancer Institute Singapore, 5 Lower Kent Ridge Road, Singapore, 119074 Singapore; 8 Assisi Hospice, 832 Thomson Road, Singapore, 574627 Singapore; 9 Office of Medical Humanities, SingHealth Medicine Academic Clinical Programme, 10 Hospital Boulevard, Singapore, 168582 Singapore; 10 Division of Supportive Care & Palliative Medicine, Department of Medicine, Alexandra Hospital NUHS, Singapore, 159964 Singapore; 11 NUS Department of Medicine, Yong Loo Lin School of Medicine, 1E, Kent Ridge Road, NUHS Tower Block, Level 10, Singapore, 119228 Singapore; 12 Medical Social Services, Singapore General Hospital, Outram Road, Singapore, 169608 Singapore; 13 Group Clinical Education, NHG Health, 1 Mandalay Road, Singapore, 308205 Singapore; 14 Institute of Humanistic Medicine, NHG Health, 1 Mandalay Road, Singapore, 308205 Singapore; 15 SingHealth Duke-NUS Medical Humanities Institute, 8 College Road, Singapore, 169857 Singapore; 16 School of Health Sciences, University of Dundee, 11 Airlie Pl, Dundee, DD1 4HJ UK; 17 Medical Affairs, National Cancer Centre Singapore, 30 Hospital Boulevard, Singapore, 168583 Singapore; 18 PalC, The Palliative Care Centre for Excellence in Research and Education, PalC c/o Dover Park Hospice, 10 Jalan Tan Tock Seng, Singapore, 308436 Singapore; 19 Palliative Care Institute Liverpool, Academic Palliative & End of Life Care Centre, Cancer Research Centre, University of Liverpool, 200 London Road, Liverpool, L3 9TA UK; 20 Health Data Science, University of Liverpool, Whelan Building, The Quadrangle, Brownlow Hill, Liverpool, L69 3GB UK; 21 Cynthia Goh Palliative Care Institute, National Cancer Centre Singapore, 30 Hospital Boulevard, Singapore, 168583 Singapore

**Keywords:** Socialization, Professional identity, Professional identity formation, Complex adaptive system, Healthcare

## Abstract

**Background:**

Professional socialization is central to professional identity formation (PIF), shaping how physicians think, act, and feel within their roles. Despite sustained scholarly attention and curricular innovation, there remains limited understanding of how professional socialization actually operates, or why individuals exposed to similar training environments develop markedly different professional identities.

**Methods:**

To examine this, we conducted a systematic scoping review using an AI-supplemented modified Systematic Evidence-Based Approach (mSEBA) to examine current theories of professional socialization. Searches across major databases and grey literature identified 46 empirical and theoretical articles, encompassing 43 theories across medicine, nursing, pharmacy, and allied health professions. A multi-stage analytic process involving thematic analysis, content analysis, and triangulation was used to synthesize findings.

**Results:**

Across included theories, professional socialization consistently emerged as a non-linear, context-sensitive process characterized by individual variation and feedback-driven adaptation. Synthesis of the literature revealed recurring patterns highlighting the importance of belief integration, identity coherence, and contextual navigation in shaping PIF. Viewed collectively, existing theories align with key features of complex adaptive systems but do not fully explain the inner processes through which individuals integrate experiences and develop stable professional identities.

**Conclusion:**

This review therefore reframes professional socialization as a belief-mediated complex adaptive process, and introduces the Krishna Theory of Socialization, an integrative framework that links system-level influences with belief-mediated identity work and a longitudinal trajectory of socialization. This theory offers a coherent account of how professional identities form, stabilize, and adapt over time, providing a conceptual foundation for future research, educational design, and learner support across health professions.

**Supplementary Information:**

The online version contains supplementary material available at https://doi.org/10.1186/s12909-026-09894-1.

## Background

Professional Identity Formation (PIF) shapes how physicians think, act, and feel in their roles, influencing patient care, interprofessional collaboration, and clinician wellbeing [1]. When professional identity is poorly formed or inadequately supported, the consequences are serious and well-documented: professionalism lapses, moral distress, burnout, and, at the extreme, attrition and suicide among trainees and practitioners [2–6]. Recognizing the importance of PIF, the Carnegie Foundation called on medical education to “explicitly cultivate the formation of professional identity” [7], and institutions have responded with increasingly sophisticated curricula, mentoring structures, and identity-focused programs [8–12]. Yet despite this sustained investment, burnout rates remain high, professional satisfaction has not meaningfully improved, and professional misconduct and physician suicide continue at troubling rates [3, 4, 13]. These outcomes suggest that current approaches, however well-intentioned, are not reaching the problem at its roots.

At the heart of PIF is the socialization process, through which individuals integrate the shared beliefs, values, principles, and practices of their professional community [14]. Through socialization, individuals are gradually introduced to the norms, roles, and expectations of their profession, learning not just what to do but who to be. Scholars have noted that socialization has been used predominantly from a functionalist perspective within health professions education, treating it as a passive process of internalizing values and norms, and have called for greater attention to the agency of individuals within the process [15]. It is in this more expansive sense, as both a structurally situated and individually mediated process, that this review uses the term. How socialization operates to shape professional identity, however, remains unclear. A better understanding of how socialization operates to impact PIF would provide opportunities to develop more effective programs and identify and support learners more meaningfully.

Current approaches to fostering PIF center on structured programs that cultivate communities of practice (CoPs), where shared norms, values, and identities are negotiated through participation in authentic work [16, 17]. In Lave and Wenger’s original formulation, a CoP is a “persistent, sustaining social network of individuals who share and develop an overlapping knowledge base, set of beliefs, values, history and experiences focused on a common practice and/or enterprise” [17]. Building on this, we conceptualize structured communities of practice (sCoPs) as formally organized, program-based groups with defined roles, responsibilities, and expectations for participation, implemented through longitudinal mentoring and educational structures in medical training [16, 18, 19]. A sCoP differs from a traditional CoP in the formal accountability and deeper commitment required of its members. These frameworks have meaningfully advanced our understanding of how environments, relationships, and curricula shape professional development, but they do not explain the internal processes through which individuals integrate these influences, nor what drives the development of a stable, coherent professional identity over time.

This limitation is not unique to CoP and sCoP frameworks. Social learning theory [20, 21] has meaningfully advanced our understanding of how observation, modelling, and participation in practice shape professional behavior and identity. Situated cognition [22] has similarly demonstrated how learning is inseparable from the contexts and communities in which it occurs, while identity-based frameworks such as figured worlds [23] have illuminated how individuals position themselves within socially and culturally constructed spaces of meaning. These contributions remain foundational. However, when applied to PIF, these frameworks attend more closely to environmental influences and developmental outcomes than to the integrative processes through which individuals make sense of and incorporate those influences into a coherent professional self. They do not fully explain non-linearity, individual variation, and context sensitivity in PIF, particularly why learners exposed to identical environments may develop markedly different professional identities.

We propose that professional socialization is better understood as a complex adaptive system (CAS) (Additional File 1), a dynamic, non-linear process in which multiple interacting agents, including learners, mentors, and institutions, co-evolve through feedback loops, adaptation, and self-organization [24]. This framing accounts for many of the features that currently puzzle educators, including why outcomes are variable, why context matters, and why professional identity cannot be engineered through curriculum alone. It does not, however, explain the inner work through which individuals interpret experiences, reconcile competing beliefs, and develop a stable professional identity over time. Understanding how this inner work operates requires attending to what individuals bring to the socialization process and how they actively engage with it.

To address this gap, we draw on the concept of personhood, defined as the constellation of belief systems across innate, individual, relational, and societal domains that together constitute “what makes you, you” [25]. As physicians encounter experiences during socialization, those experiences may resonate with or challenge existing beliefs across these domains. Through sensitivity to dissonance, judgment about significance, willingness to engage, and efforts to maintain coherence, individuals undertake identity work, the conscious and unconscious adjustments that shape who they are becoming as professionals [1]. Over time, this identity work consolidates into an internal compass, a stabilized sense of professional self that guides interpretation and action even in unfamiliar situations [26]. Viewing socialization as a CAS mediated through personhood and the development of an internal compass offers a more integrative account of how professional identity forms, one that explains not just what shapes the individual from without but how the individual actively shapes themselves from within.

To examine whether this integrative account is supported across the existing literature, we conducted a systematic scoping review of current theories of professional socialization among healthcare professionals. The findings inform the development of a framework that can meaningfully guide how educators design programs, support learners, and understand the process they are trying to cultivate.

## Methods

### Study design and reporting

We conducted a systematic scoping review using an artificial intelligence-supplemented adaptation of the modified Systematic Evidence-Based Approach (mSEBA). mSEBA is an established methodology that has been applied across peer-reviewed studies examining PIF, personhood, and related educational phenomena [11, 27, 28]. The approach integrates AI-assisted searching and analysis with concurrent manual review, supported by an expert team to enhance transparency, rigor, and reproducibility while maintaining human oversight throughout the review process.

This review was conducted and reported in accordance with the PRISMA Extension for Scoping Reviews (PRISMA-ScR) [29], appended in Additional File 2.

### Overview of the mSEBA process

**Fig. 1:**
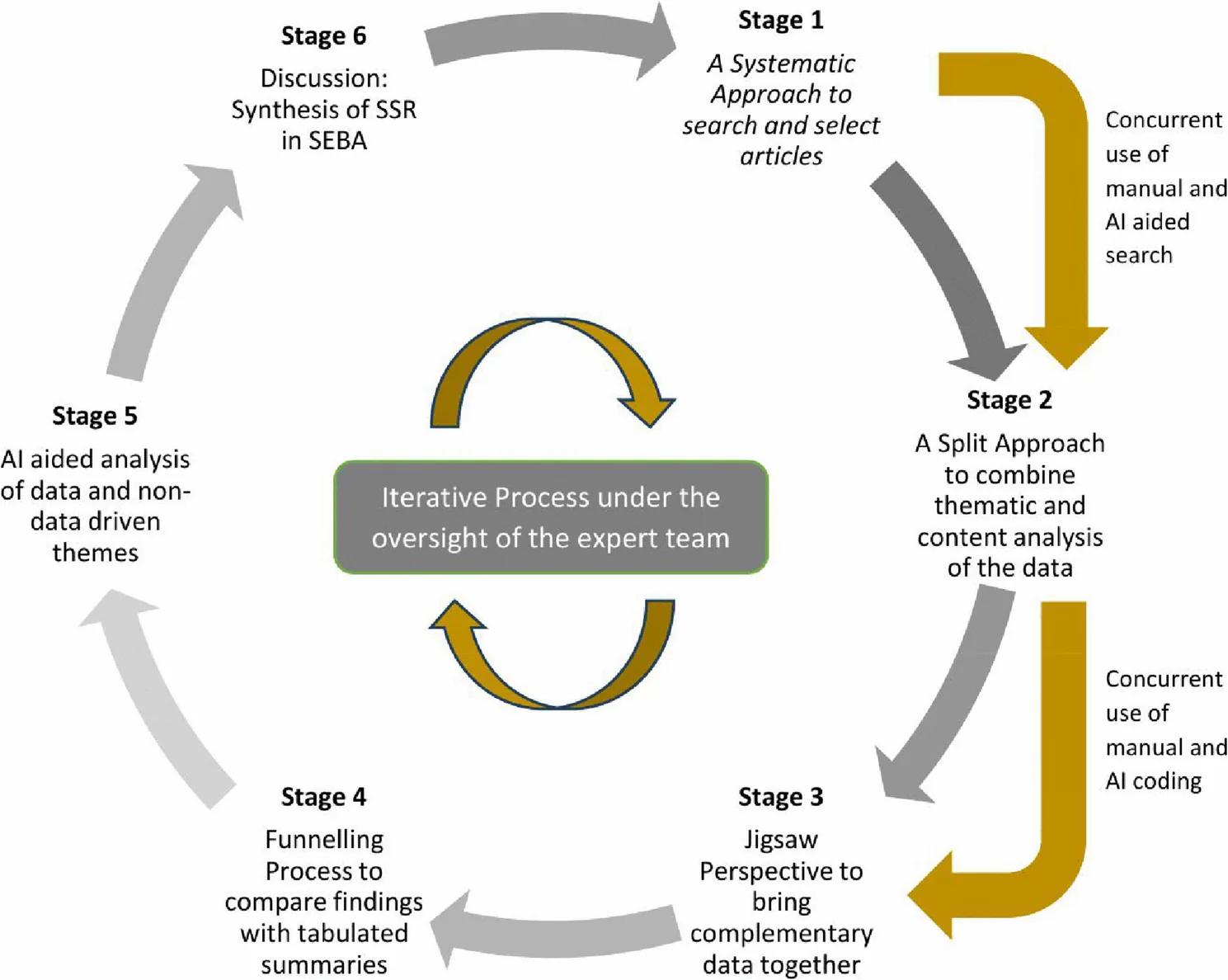
Stages of the modified Systematic Evidence-Based Approach [30]

The stages of the mSEBA methodology used in this review are summarized in Fig. 1. The process comprises six sequential but iterative stages: a systematic search and selection of articles, a Split Approach combining thematic and content analysis, a Jigsaw Perspective integrating findings across analyses, a Funneling Process consolidating higher-order categories, an AI-aided comparison of data-driven and non-data-driven literature, and a final synthesis. Throughout all stages, the process was conducted under the oversight of an expert team, with movement between stages informed by emerging findings and ongoing review.

### Research question and eligibility criteria

This review addressed the research question: “*What is known of current theories of socialization among healthcare professionals*?” This broad framing is consistent with scoping review methodology, which is designed to map the landscape of existing literature across a field characterized by conceptual heterogeneity, rather than to test specific hypotheses or evaluate discrete interventions [31]. The question is deliberately focused on theories of socialization within healthcare professional education, excluding broader socialization literature that does not engage with theoretical frameworks. Eligibility criteria were defined using a Population–Concept–Context (PCC) framework. The population comprised healthcare professionals across disciplines. The concept focused on theories of socialization and critical analyses of socialization processes. The context examined the interplay between socialization and professional identity, including processes, applications, benefits, challenges, and limitations. Full inclusion and exclusion criteria are presented in Table 1.

**Table 1:** Population, Concept and Context (PCC) framework, study design and inclusion and exclusion criteria applied to database search

PCC	Inclusion Criteria	Exclusion Criteria
Population	Healthcare professionals across all disciplines	No specified population
Concept	Critical analysis of theories of socialization Critical analysis of elements of socialization	Studies without focus on theories of socialization Studies without focus on critical analysis of socialization
Context	The interplay between socialization and professional identity • Processes • Applications • Benefits • Challenges • Limitations	Studies without focus on the interplay of socialization with professional identity
Study Design	Articles in English or translated to English Published within 1^st^ January 2000 and 31^st^ July 2025 Any study type Any publication type Databases used: Scopus, ProQuest Education Database, ProQuest Social Sciences Database, ProQuest Sociology Database, PubMed, ERIC, CINAHL, PsycINFO, Embase AI platforms used: ChatGPT5pro, Manus AI	Non-English language

The stages below describe how evidence was identified, analyzed, and synthesized; conceptual interpretation is presented in the Results and Discussion.

### Stage 1 of mSEBA: a systematic approach to search and select articles

An expert team comprising medical educators, researchers and clinicians guided the research team through all stages of the review. The expert team provided oversight, supported adherence to PRISMA-ScR reporting standards, and served as the human oversight mechanism within the AI-supplemented workflow. Both manual and AI-assisted searches were conducted concurrently.

#### Manual searches

Manual searches were performed independently by two reviewers and overseen by a senior mentor as part of a structured research mentoring program. Independent database searches were conducted between September 12 and September 20, 2025, across Scopus, PubMed, Embase, ERIC, CINAHL, PsycINFO, ProQuest Education Database, ProQuest Social Sciences Database and ProQuest Sociology Database. Searches were limited to articles published in English between January 1, 2000 and July 31, 2025.

#### AI-assisted searches

In parallel, AI platforms, including ChatGPT-5 Pro and Manus AI, were used by independent researchers to apply the same search protocols. Results from AI-assisted searches were cross-checked against the manually identified articles by two independent reviewers. Articles identified exclusively through AI-assisted searches were subjected to the same full-text screening process applied to manually retrieved articles, with inclusion confirmed against the predefined PCC criteria. Discrepancies between reviewers were resolved through discussion, with unresolved cases referred to a senior team member. The full search strategy is provided in Additional File 3. A total of 50 articles were identified and all AI-assisted outputs were reviewed and validated by the research team. 31 articles overlapped with the articles identified from the manual searches, leaving 19 articles identified from the AI-assisted searches. Eventually, 16 eligible articles were included in the study after undergoing full-text reviews. AI tools were used to assist, not replace, human judgment, and no AI-identified article was included without independent verification against the eligibility criteria.

#### Iterative process

An iterative process was employed throughout the searches. Initial searches focused on physicians; however, both manual and AI-assisted searches did not yield substantive findings within the literature. Notably, the articles identified in initial searches featured mixed populations, and relevant findings focused on other healthcare professionals. The limited data on solely physicians necessitated the expansion of the population to include all healthcare professionals, with corresponding refinement of the PCC framework. This iterative process allowed the research team to refine the proceeding searches in accordance with our initial findings, broadening the scope of the review to include a greater range of data from eligible articles.

### Stage 2 of mSEBA: the split approach

Included articles underwent concurrent thematic and content analyses conducted by independent teams. Qualitative evidence and quotations from the articles were identified using the codes and categories derived from Hunter et al.’s article entitled, ‘Becoming a Scientist: The Role of Undergraduate Research in Students’ Cognitive, Personal, and Professional Development’ [32]. ChatGPT5Pro, Co-Pilot and NotebookLM were used to support coding consistency and to verify patterns identified through manual analysis, without replacing human interpretation. A member of the research team (LKRK) then uploaded the included articles to each AI platform. Each AI platform was prompted to identify the common themes and patterns across all articles using the codes and categories from Hunter et al.’s article [32]. The summary of codes generated by each AI platform (Additional File 5) were then compared by the research team to further refine the categories previously identified.

Thematic analysis explored patterns and meanings within the data using Braun and Clarke’s [33] approach, while content analysis focused on identifying and categorizing explicit theoretical elements related to socialization using Hsieh and Shannon’s [34] directed content analysis approach.

The Split Approach strengthens the trustworthiness of the synthesis by pairing thematic analysis with content analysis as complementary methods. Thematic analysis allows for the interpretive depth necessary to explore qualitative concepts with sufficient nuance [33], whereas content analysis offers a structured approach to review and categorize the dataset [34]. Hence, the Split Approach offers a more holistic and robust review through complementary methods of analysis by ensuring analytic rigor without compromising the value of subjective meaning-making. The findings were verified by the research team and discrepancies were resolved through team discussion.

### Stage 3 of mSEBA: the Jigsaw Perspective

Findings from the thematic and content analyses were integrated using the Jigsaw Perspective. This stage centered on the notion that complementary qualitative data gives “a richer, more nuanced understanding of a given phenomenon” [33]. Following this, complementary data was merged according to Phases 4 to 6 in France et al.’s adaptation [35] of Noblit et al.’s seven-phase meta-ethnographic approach [36]. Phase 4 involves determining how studies are related, Phase 5 focuses on translating the interpretations from studies into one another and synthesizing the findings. Phase 6 characterizes the line of argument within the synthesis [35]. Adapting these principles of meta-ethnography, themes and categories were treated as complementary pieces of evidence and iteratively combined to generate a more comprehensive understanding of the socialization process. This stage was conducted by senior researchers to support synthesis across diverse sources.

### Stage 4 of mSEBA: the funneling process

The merged findings were compared with tabulated summaries (Additional File 4) and AI-assisted summaries (Additional File 5) to refine and consolidate the synthesis, yielding higher-order analytic categories that informed the subsequent organization of the results.

### Stage 5 of mSEBA: AI-aided analysis of data- and non-data-driven literature

Using AI-assisted analysis, themes derived from empirical studies were compared with those identified in non-data-driven theoretical literature, including theoretical, conceptual, narrative, and perspective-based publications which aim to advance frameworks and highlight interpretive accounts. Non-data-driven literature was included to enhance theoretical coherence and avoid privileging empirical data, ensuring that empirical findings are grounded in conceptual frameworks [31]. This stage evaluated convergence between data-driven and conceptual accounts and assessed the potential influence of theoretical bias on the synthesis. Consistency across these sources supported the robustness of the identified patterns.

### Stage 6 of mSEBA: discussion and synthesis of the SSR

The final stage involved synthesis of the findings across all stages to develop an integrated account of socialization processes within healthcare professions. This synthesis informed the interpretive and conceptual contributions presented in the Discussion.

#### Reflexivity and AI transparency

The research team comprised medical students and research assistants supported by clinicians with expertise in internal medicine, palliative care, medical education, qualitative research, and systematic reviews. The medical students were members of a peer-mentorship research program; their personal experiences influenced the study design and data interpretation. Reflexivity refers to the deliberate examination of each researchers’ professional roles, prior experiences, and perspectives which may shape analysis and data interpretation [37]. The teams engaged in personal and group reflexivity throughout the process to minimize the impact of personal experience bias and enhance analytic rigor. To provide a balanced review, an expert team comprising of local educational experts and clinicians at National Cancer Centre Singapore, Palliative Care Institute Liverpool, Alexandra Hospital NUHS, NHG Health and Duke-NUS Medical School guided the mSEBA process.

AI tools were used to assist data organization, coding verification, and synthesis, but all analytic decisions remained under human oversight.

## Results

### Overview of included studies

**Fig. 2:**
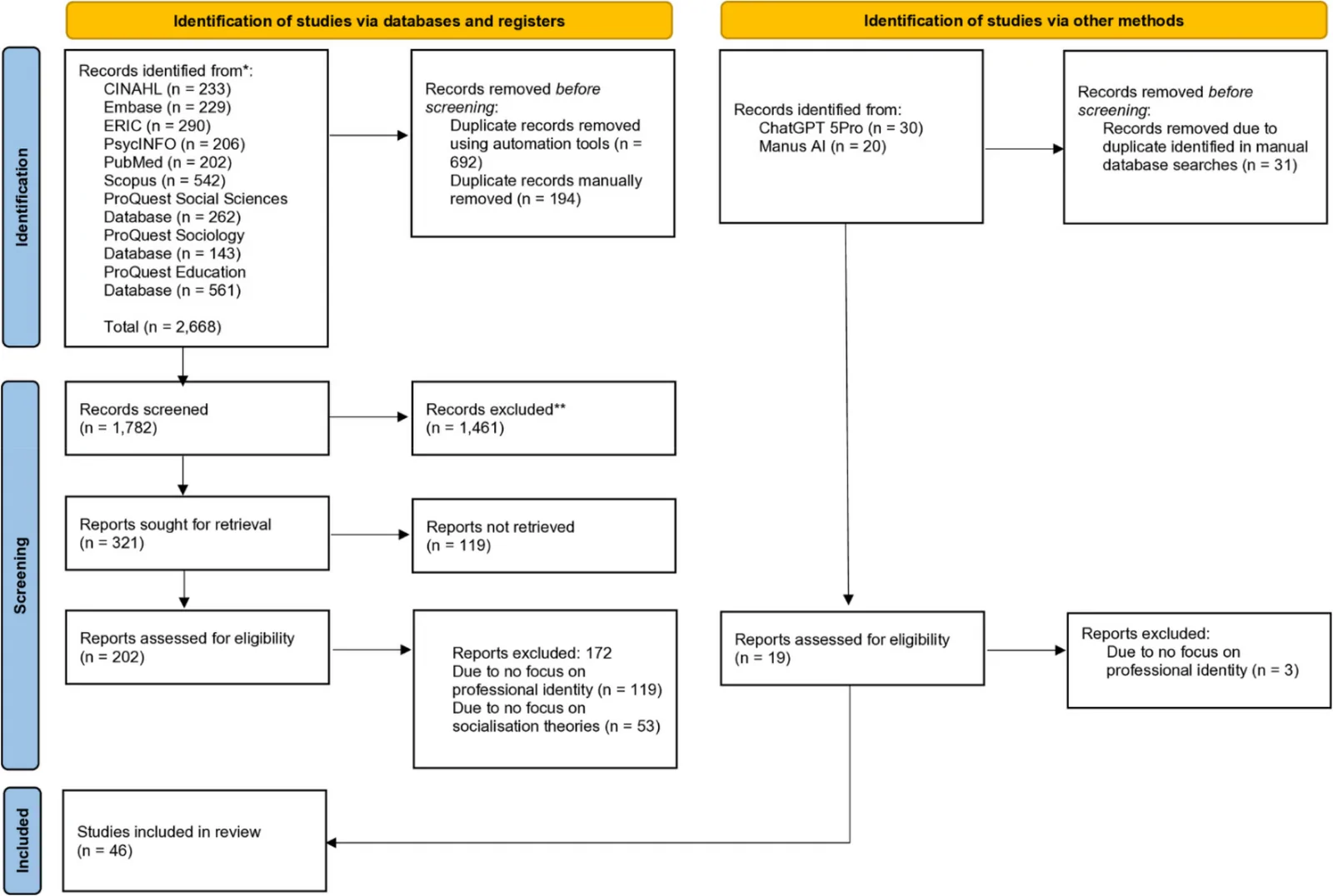
PRISMA flowchart

Database searches identified 2,668 articles. Following screening and full-text review, 46 articles were included in the final synthesis (Fig. 2).

Included studies spanned medicine, nursing, pharmacy, dentistry, physiotherapy, and allied health professions, as well as the fields of education and psychology (Table 2). Studies employed diverse methodologies, including qualitative, quantitative, and mixed-methods approaches, and addressed socialization across undergraduate, postgraduate, and continuing professional development contexts.

**Table 2:** Fields of included articles

Domain	Subset	References
Healthcare Professionals	Medicine	[38–42]
	Nursing	[38, 40, 43]
	Pharmacy	[38, 44–47]
	Dentistry	[38]
	Physiotherapy	[48]
	Unspecified	[14, 21, 49–51]
Healthcare Students	Medicine	[13, 20, 22, 23, 52–63]
	Nursing	[22, 64, 65]
	Athletic Training	[66]
Healthcare, Education	Science	[32, 67]
	Psychology	[68–70]
	Teaching and Education	[71–76]

### Synthesis-level patterns across theories of socialization

Synthesis of the 43 socialization theories identified across the 46 included articles (Supplementary File 3) revealed consistent patterns that transcend disciplinary and methodological boundaries. Rather than discrete or competing frameworks, these theories collectively point toward shared underlying features of how professional socialization operates. The analytic outputs of the review were subsequently reorganized into the synthesis-level patterns below, which describe how professional socialization operates across contexts and individuals. These patterns cut across individual theories and reflect recurring features of socialization as a phenomenon through the lens of CAS, rather than discrete analytic categories.

### Non-linearity and individual variation

Socialization does not follow a uniform trajectory; individuals within the same training environment, exposed to identical curricula and mentors, develop markedly different professional identities [52, 53]. Progression from peripheral to central participation in a sCoP is neither straightforward nor consistent. Trajectories are shaped by individual narratives, personal histories [71, 73], motivations, confidence, and reflective capacity [56], as well as differences in perceived competence and readiness for responsibility [54]. This variability challenges linear models of professional development and suggests that socialization is mediated by individual-level factors that shape how environmental influences are interpreted and enacted.

### Feedback loops and identity coherence

Professional socialization involves continuous cycles of experience, reflection, and adaptation [63]. Individuals encounter experiences that either resonate with or challenge existing beliefs. Such encounters prompt sensitivity to dissonance and judgments about significance [46] as well as willingness to engage and efforts to maintain coherence between emerging professional values and pre-existing personal beliefs [13, 75]. Repeated exposure to resonant beliefs consolidates certain values, strengthening some belief systems over time, while reflective practices support reinterpretation of earlier experiences in light of growing insight and competence [63]. Together, these feedback-driven processes shape identity work iteratively rather than additively.

### Context-sensitivity and social validation

The significance of any given experience varies depending on temporal positioning within training [41] as well as environmental and relational conditions within specific sociocultural and institutional contexts [42, 72, 73]. Hidden curricula influence how professional norms are tacitly learned and internalized [57], while sociocultural expectations shape what forms of professional behavior are valued or sanctioned [23]. Institutional hierarchy further mediates how individuals interpret authority, feedback, and legitimacy [71, 72] and the quality of mentoring relationships affects whether experiences are perceived as supportive or threatening [66]. Identity formation is additionally shaped by social validation, whereby emerging professional identities are recognized and affirmed by the sCoP [52, 66, 74]. Broader sociocultural influences, including collectivist values, family-centric norms, and institutional culture, further shape how individuals negotiate balance across personal, relational, and professional commitments [71, 76].

### Belief integration across domains of personhood

Socialization involves integrating professional expectations with existing belief systems across multiple domains of personhood, including innate characteristics (spiritual, existential, and demographic), individual attributes (emotions, behaviors, and personality), relational commitments (family and friendships), and societal roles (professional, legal, and ethical norms). Integrating professional expectations requires navigating tensions between competing ethical and cultural values [55] while sustained PIF depends on reflective processes that support coherence between personal and professional selves [44]. Transformative experiences and disorienting moments can serve as critical junctures that challenge existing beliefs [43] while sources of moral strain may catalyze shifts in professional self-concept over time [65].

### Development of an internal compass

The consolidation of enrooted beliefs and increasing identity coherence give rise to an internal compass over time, a stabilized sense of professional self that guides interpretation and action even in unfamiliar situations [47]. This internal compass reflects the cumulative effect of iterative identity work, enabling individuals to respond to novel challenges in ways that remain consistent with their integrated professional and personal identities. Its development is supported by temporal dissociation, in which earlier experiences are re-evaluated in light of new insights [60], as well as socially mediated reflective cycles, including debriefs and learning from role models, that shape how experiences are interpreted and integrated into professional identity [20].

### Theoretical lens

Framing socialization as a CAS explains why it resists reduction to simple cause-and-effect models. However, this perspective characterizes the system rather than the inner work through which individuals integrate experiences, reconcile beliefs across domains of personhood, and develop a stabilized professional self over time. To address this gap, we propose the Krishna Theory of Socialization, an organizing framework that emerged from this synthesis and translates these empirically derived patterns into a structured, longitudinal model of PIF.

### The Krishna Theory of Socialization

At its core, the Krishna Theory of Socialization (KToS) comprises two complementary components: a mechanistic model that explains how individuals integrate experiences and beliefs at the level of personhood, and the Socialization Process, a longitudinal trajectory that describes how this identity work unfolds over time within a sCoP.

#### Mechanism of identity work: the Krishna-Pisupati Model of Identity Formation

**Fig. 3:**
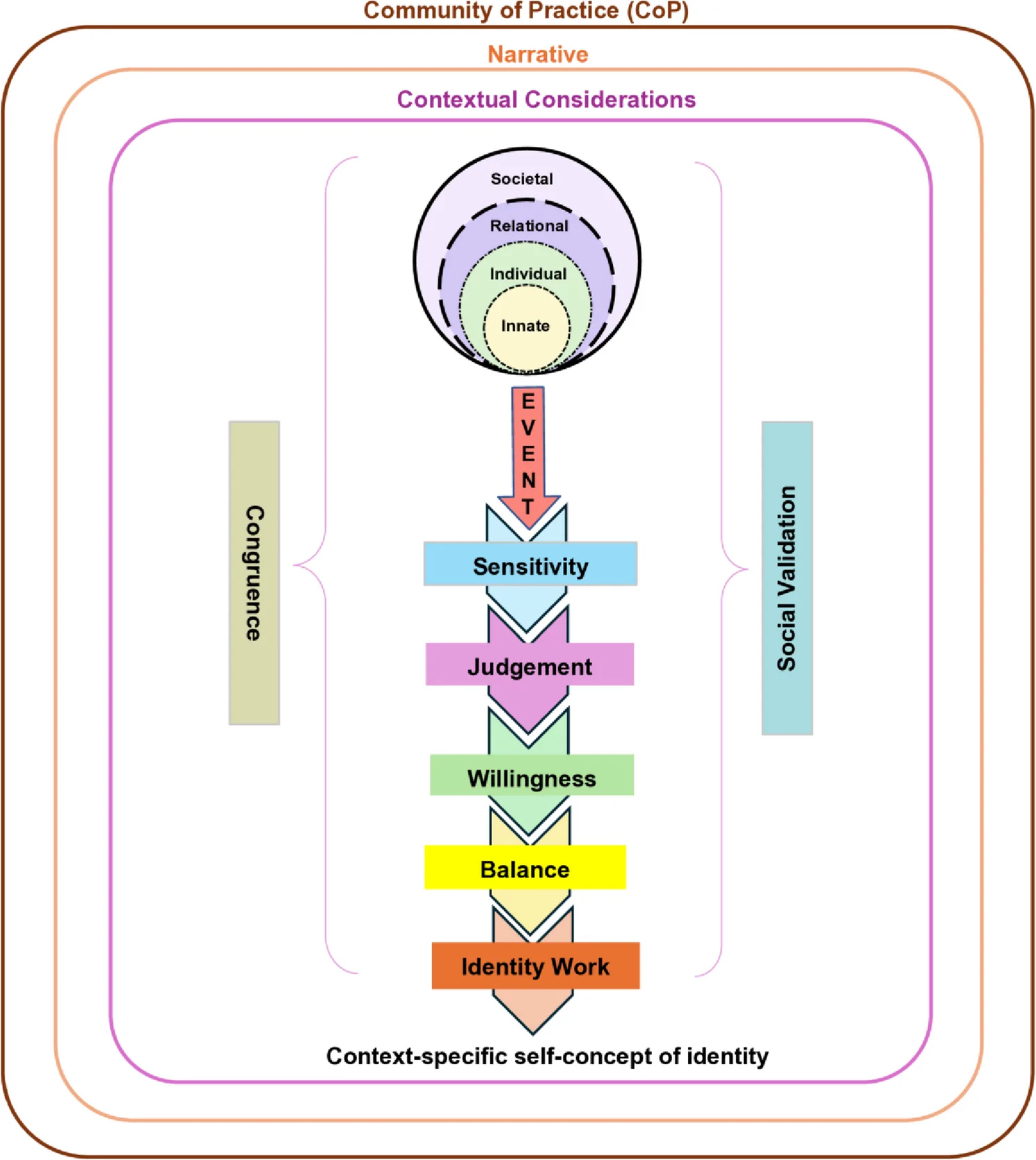
The Krishna-Pisupati Model of Identity Formation

Figure 3 illustrates the Krishna-Pisupati Model of Identity Formation (KPM), depicting how individuals integrate professional experiences with existing belief systems. The concentric rings at the top of the figure represent the four domains of personhood — innate, individual, relational, and societal — reflecting the layered belief systems and values that constitute an individual’s sense of self. When a professional experience is encountered, it may resonate with or generate dissonance across these domains, activating the identity work processes depicted below. These proceed as a sequential series of interrelated steps: sensitivity to dissonance, judgment regarding the significance of the experience, willingness to engage with competing values, efforts to achieve balance across domains, and ongoing identity work. Throughout this sequence, congruence and social validation within the sCoP operate as moderating influences, shaping how experiences are interpreted and integrated. Over time, this process gives rise to a more stable internal compass and a context-specific self-concept of identity, situated always within the broader community of practice.

#### Longitudinal trajectory of socialization

**Fig. 4:**
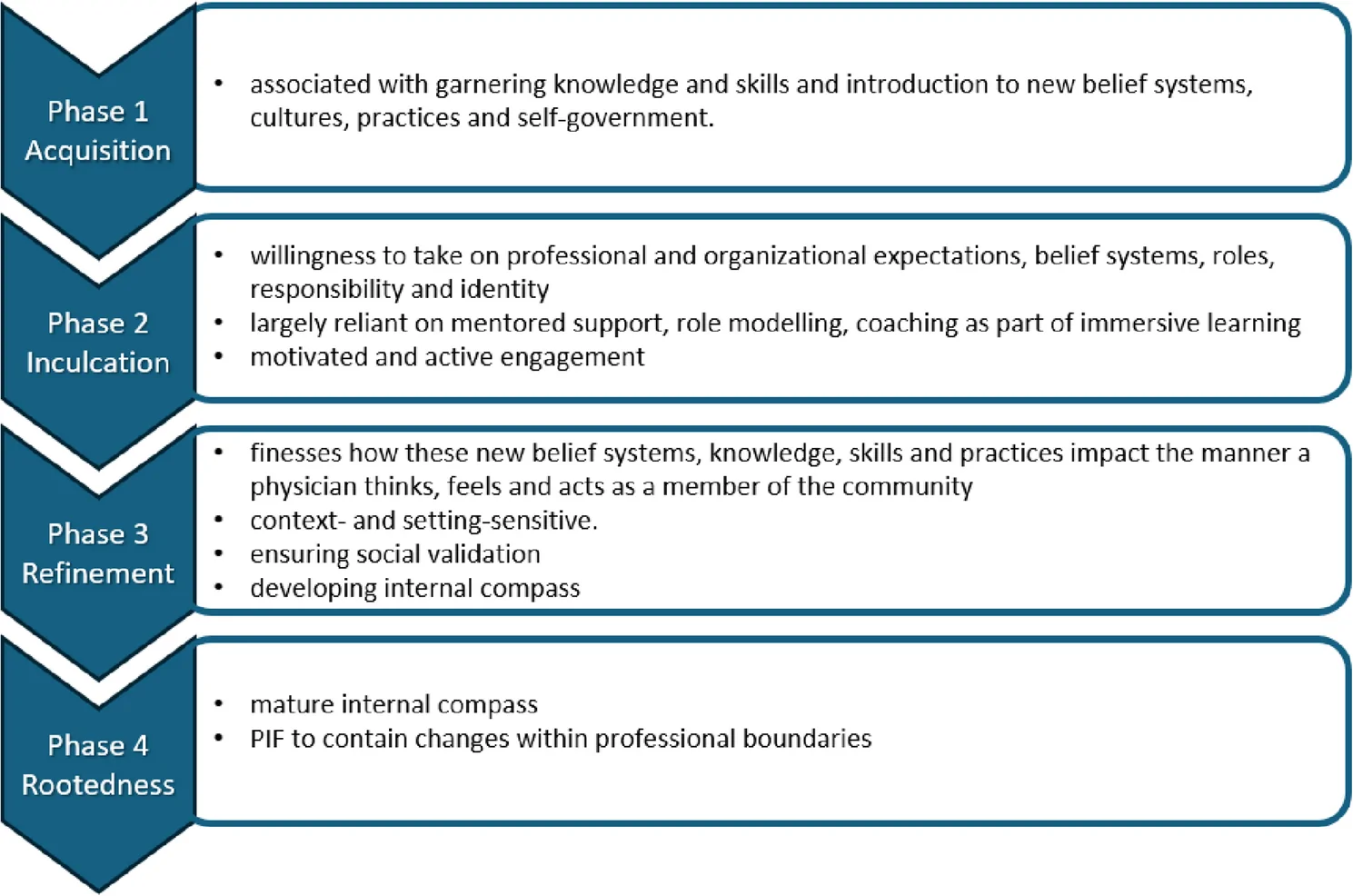
The socialization process

The second component of the KToS is the Socialization Process, a longitudinal but non-linear trajectory depicting how identity work unfolds iteratively across professional development through four broad phases: acquisition, inculcation, refinement, and rootedness (Fig. 4).

During acquisition, individuals are introduced to new knowledge, skills, norms, and belief systems associated with professional practice. During inculcation, learners increasingly engage with professional expectations through guided participation, mentoring, and role modeling within structured communities of practice. Refinement involves more nuanced, context-sensitive identity work, as individuals calibrate their beliefs, behaviors, and professional self-concept while seeking social validation and coherence. Rootedness reflects the maturation of an internal compass, enabling individuals to contain change within professional boundaries while maintaining identity stability in the face of novel or challenging circumstances.

These phases are not strictly sequential. Rather, they represent recurring patterns of socialization that may be revisited as individuals encounter new roles, contexts, or professional challenges.

#### An integrated theory of professional socialization

Together, the KPM and the Socialization Process operationalize professional socialization as a CAS at the individual level. The KToS explains how dynamic, context-sensitive system-level influences are mediated through belief integration and identity work, resulting in the gradual stabilization of professional identity over time.

By integrating this mechanism with trajectory, the theory provides a structured yet flexible framework for examining professional socialization across health professions and training stages. It offers a conceptual foundation for educational design, faculty development, and learner support by making visible the processes through which professional identities are formed, challenged, and sustained within sCoPs.

**Fig. 5:**
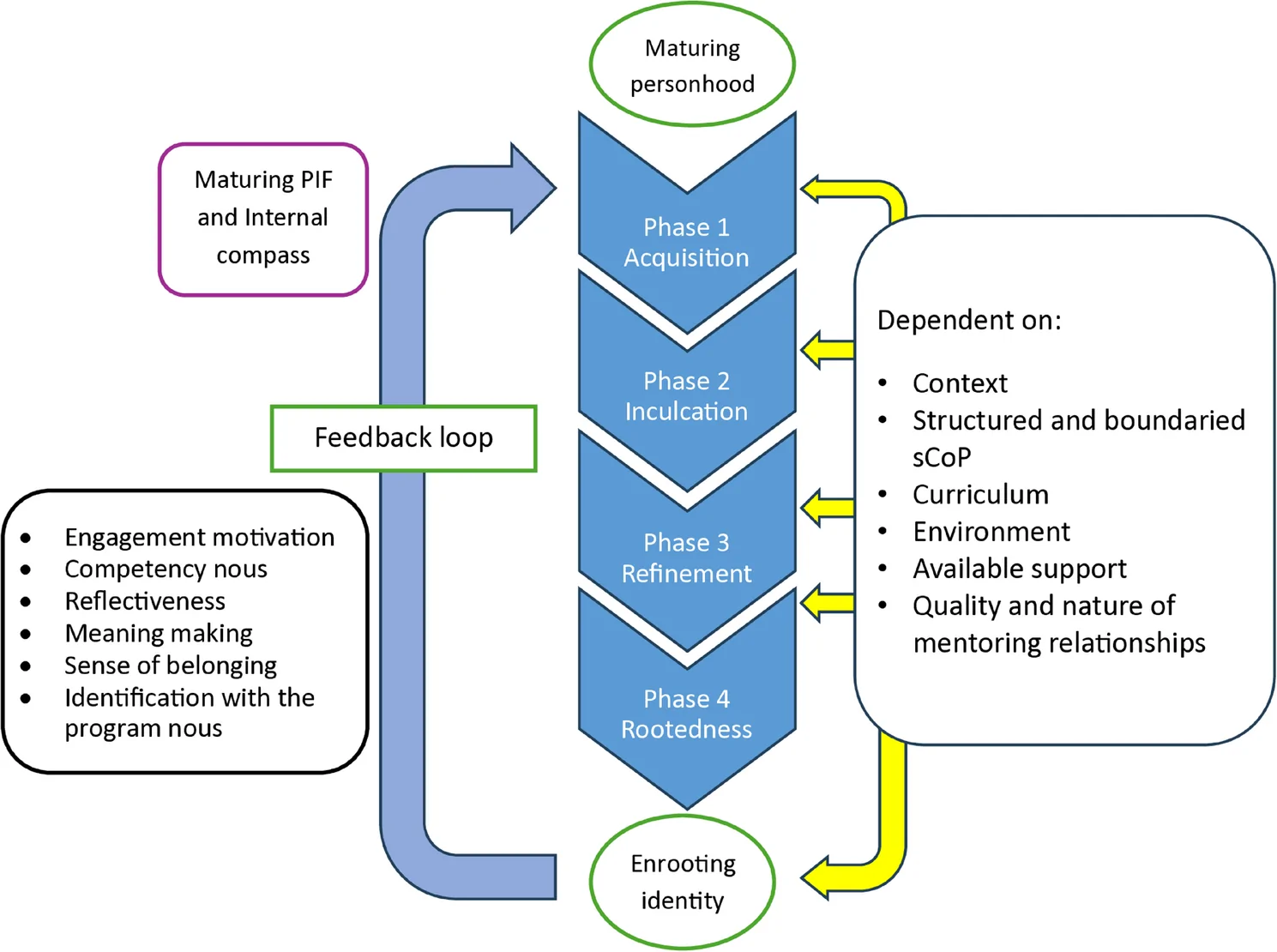
The integrated representation of the socialization process as articulated by the Krishna Theory of Socialization

Figure 5 presents an integrated representation of the Socialization Process as articulated by the KToS, building on the mechanistic processes described in the KPM and the four-phase longitudinal trajectory. Maturing personhood, depicted at the top, represents what each individual brings into the socialization process, while enrooting identity, at the bottom, reflects the cumulative outcome of a stabilized professional self progressively embedded within the sCoP. Progression through the four central phases of acquisition, inculcation, refinement, and rootedness is shaped by the contextual conditions depicted on the right, including curriculum, environment, mentoring relationships, and available support. The individual characteristics on the left reflect the internal resources each learner brings to and develops through the process, and the feedback loop illustrates how the outcome of maturing PIF and internal compass feeds back into the process, making socialization self-reinforcing and iterative rather than terminal.

## Discussion

The findings of this review point toward a coherent reframing of professional socialization, one that moves beyond describing what shapes the individual from without, toward explaining how the individual actively integrates those influences from within. The synthesis-level patterns identified across 43 theories collectively reflect the hallmarks of a CAS, in which multiple interacting agents co-evolve through non-linear feedback loops [24] and give rise to emergent properties such as professional identity [61]. Together, they describe a dynamic, interdependent system in which individual and environmental factors continuously shape and reshape one another.

### Principal findings and conceptual contribution

This SSR addressed a persistent problem in medical education; despite decades of scholarship on professional socialization, we still lack an integrative account of how healthcare professionals actually develop professional identities. While existing theories describe environmental influences, social learning processes [20] and participatory trajectories within an sCoP [16], they do not explain why individuals exposed to similar training environments develop markedly different professional identities, nor how these identities achieve stability over time.

Synthesizing 43 theories across 46 empirical and theoretical studies revealed consistent patterns that transcend disciplinary boundaries. Socialization does not proceed along a uniform or linear trajectory; instead, individuals within the same curriculum and community may experience divergent professional identity outcomes [52, 53]. These differences reflect continuous feedback loops between experience and belief systems and mediated through structured reflection and feedback processes [63]. Importantly, how these experiences are interpreted is highly sensitive to context, including hidden curricula and implicit institutional norms [57], particularly those conveyed through assessment and feedback practices [23], institutional hierarchies [53] and the quality of mentoring relationships.

Across the reviewed literature, PIF emerged as a belief-mediated process. Experiences encountered during training are interpreted through existing belief systems across multiple domains of personhood [25]. Over time, repeated engagement with resonant beliefs supports the development of an internal compass, a stabilized sense of professional self that guides judgment and action even in unfamiliar or challenging situations [26].

Taken together, these findings support a reframing of professional socialization as a CAS through multiple interacting agents who co-evolve through feedback loops, giving rise to emergent professional identities that cannot be predicted from individual components alone [24]. Recent applications of complex systems frameworks to conceptualize PIF further underscore how contextual variation and individual sense-making shape these emergent outcomes [61]. However, while this framing captures the dynamics of the system, it does not explain the inner work through which individuals reconcile competing beliefs and develop stable professional identities over time.

The core conceptual contribution of this review is therefore twofold. Firstly, we demonstrate that personhood, understood as the integration of beliefs across innate, individual, relational, and societal domains, provides a mechanism through which system-level influences are interpreted and enacted at the individual level [25]. Secondly, we show that the development of an internal compass explains how ongoing identity work leads to coherence and continuity across contexts and career stages, including through the reinterpretation of earlier experiences in light of new professional understanding [60].

The KToS integrates these insights into a unified framework comprising two complementary components: a mechanistic model of belief-mediated identity work, articulated through the Krishna–Pisupati Model, and a longitudinal trajectory of socialization described as the Socialization Process. By making explicit the integrative processes that prior models left implicit, it offers a structured yet flexible account of how professional identities form, stabilize, and adapt within a sCoP [1, 24].

### Advancing theory: professional socialization as a belief-mediated CAS

Existing theories of professional socialization have made important contributions by elucidating how learning occurs through participation in a sCoP, observation of role models, and progressive engagement with professional norms and expectations [16, 20, 21]. These perspectives have been particularly effective in explaining how environments, relationships, and curricula shape professional development. However, they tend to privilege external structures and interactions, offering limited insight into how individuals internally interpret, integrate, or resist these influences over time [1].

Eteläpelto and colleagues argue that professional identity is actively negotiated through individual agency, shaped by the interplay between personal history, values, and sociocultural conditions [77, 78]. Similarly, Billett (2001) demonstrates that identical workplace environments produce different learning outcomes because individuals bring different dispositions, intentions, and histories to what those environments afford [79].

By integrating CAS thinking with a belief-mediated account of identity work, the KToS advances existing frameworks without displacing them. sCoPs, social learning, and role modelling remain central influences, but their effects are understood as contingent on how individuals integrate these experiences within their evolving sense of personhood. This integration clarifies why professional socialization is simultaneously structured and unpredictable, shaped by common environments yet expressed through highly individualized professional identities.

### Mechanism and trajectory: integrating belief-mediated identity work with longitudinal socialization

While CAS framing explains the dynamic and non-linear nature of professional socialization, it does not specify how individuals internally process experiences within such systems. The KToS addresses this by proposing that internal processing occurs through belief-mediated identity work operating within a longitudinal trajectory of socialization, together providing both a mechanism and a developmental arc that CAS framing alone cannot supply.

Belief-mediated identity work is activated when experiences resonate with or challenge existing beliefs. Encounters that align with established beliefs may reinforce aspects of professional identity, whereas dissonant experiences prompt reflection, re-evaluation, and potential recalibration of beliefs [1]. Over time, repeated cycles of experience, reflection, and adjustment contribute to the consolidation of certain beliefs, supporting greater coherence between personal values and professional roles [63]. Temporal reinterpretation of earlier experiences plays a key role in this process, as individuals reframe past challenges and successes in light of evolving professional understanding [60].

Integrating mechanism and trajectory clarifies how professional socialization can be both adaptive and stabilizing. Belief-mediated identity work allows individuals to respond flexibly to contextual variation, while the gradual development of an internal compass supports coherence and durability of professional identity across career stages. Together, these processes explain how professional socialization produces identities that are shaped by common environments yet expressed in highly individualized ways.

### Educational implications

Understanding professional socialization as a belief-mediated complex adaptive process has important implications for how medical education is designed, delivered, and supported. Rather than viewing PIF as a predictable outcome of exposure to curricula or role models, this framing highlights the need to attend to how learners interpret experiences, negotiate dissonance, and integrate beliefs over time.

### Curriculum design

Curricula that aim to support PIF should move beyond providing standardized experiences toward intentionally creating opportunities for reflection, sense-making, and belief integration. While participation in authentic clinical contexts and sCoP remains essential [16], exposure alone is insufficient to ensure coherent identity development. Structured opportunities to engage with moments of uncertainty, moral tension, and role transition can help learners articulate and examine how professional expectations interact with existing beliefs [1]. Designing curricula that acknowledge non-linearity and allow learners to revisit identity work across training stages may better support longitudinal professional development.

### Faculty development

Faculty plays a critical role in shaping how learners interpret and respond to professional experiences. Beyond serving as role models, supervisors and mentors can function as guides who help learners recognize dissonance, reflect on its significance, and navigate competing professional and personal values [18, 20]. Faculty development initiatives that enhance awareness of belief-mediated identity work may enable educators to better support learners during periods of identity disruption, rather than interpreting such moments solely as deficits or performance concerns.

### Learner support

Recognizing that PIF is iterative and individually variable has important implications for learner support systems. Periods of uncertainty, alienation, or identity destabilization should be understood as expected features of professional socialization rather than as signs of failure, particularly when learners experience exclusion or marginalization within clinical environments [65]. At the same time, disorienting moments that arise during role transition can serve as productive developmental junctures when they are accompanied by recognition, participation, and legitimation within communities of practice, supporting the consolidation of professional identity over time [43]. Support structures that normalize these experiences and provide space for guided reflection may help learners integrate challenging experiences into a coherent professional identity. Over time, such support may contribute to the development of an internal compass that enables learners to navigate future challenges with greater confidence and stability [26].

Across these domains, a shared principle emerges. Supporting PIF requires moving beyond standardized exposure toward deliberate attention to how individuals interpret, integrate, and make meaning of their professional experiences over time.

### Methodological contributions and directions for future research

In addition to its theoretical contributions, this review also demonstrates the value of synthesis-level analysis for theory development in PIF. Rather than aggregating outcomes or testing predefined hypotheses, the use of a systematic scoping approach enabled the identification of recurring patterns across diverse theoretical traditions, professional groups, and educational contexts. This approach was particularly suited to a field characterized by conceptual fragmentation and heterogeneous methodologies.

The application of the modified Systematic Evidence-Based Approach (mSEBA) supported this synthesis by integrating complementary analytic strategies, including thematic analysis, content analysis, and iterative triangulation. This pluralist approach [37] enabled the consolidation of both empirical findings and conceptual arguments, while preserving the interpretive depth required to examine complex phenomena such as professional socialization. Importantly, the inclusion of human oversight alongside AI-supported analysis strengthened transparency and rigor, rather than replacing interpretive judgment.

The findings of this review also highlight several priorities for future research. Firstly, there is a need for empirical studies that explicitly examine belief-mediated identity work, particularly how individuals integrate experiences across domains of personhood over time. Longitudinal qualitative designs may be especially valuable for examining how identity destabilization, disorienting moments, and legitimation contribute to the development of an internal compass and a stable professional self.

Secondly, future work should explore how the KToS operates across different sociocultural and institutional contexts. Many theories of PIF have been developed in Western training environments, yet the synthesis presented here suggests that hierarchy, collectivist values, and contextual norms play a significant role in shaping identity trajectories. Comparative and cross-cultural studies could help refine and extend the theory.

Finally, the KToS provides a conceptual framework that can guide hypothesis generation and empirical testing, rather than serving as a prescriptive model. Future research might examine how specific educational practices influence belief integration, how internal compass development can be supported during transitions, and how identity coherence relates to professional well-being and ethical practice over time.

### Limitations

Several limitations should be considered when interpreting the findings of this review. Firstly, as a SSR, this work aimed to map and synthesize existing theories of professional socialization rather than to evaluate the quality or strength of evidence supporting individual models. Consequently, the conclusions drawn reflect patterns across the literature rather than empirical validation of specific causal mechanisms.

Secondly, the synthesis is necessarily shaped by the scope and characteristics of the included literature. Although the review encompassed a wide range of health professions, training stages, and methodological approaches, the theories examined are drawn primarily from published English-language sources. As such, perspectives from non-English or unpublished scholarship may be underrepresented, and certain sociocultural contexts may not be fully captured.

Thirdly, the integrative nature of the synthesis involves interpretive judgment. While the mSEBA and triangulation processes were employed to enhance transparency and rigor, the identification of synthesis-level patterns and the articulation of the KToS reflect analytic decisions made by the research team. Alternative syntheses or theoretical interpretations may be possible.

Finally, the KToS is a conceptual framework derived from synthesis rather than empirical testing. While it provides a coherent account of professional socialization grounded in existing literature, further empirical work is needed to examine its applicability, explanatory power, and utility across different educational and clinical contexts.

## Conclusion

Professional socialization has long been recognized as central to the formation of healthcare professionals’ identities yet existing theories have struggled to account for the variability, non-linearity, and internal coherence observed across individuals and contexts. Through a systematic scoping synthesis of contemporary theories, this review demonstrates that professional socialization operates as a complex adaptive process in which identity formation is mediated through belief integration and sustained over time by the development of an internal compass.

The KToS offers an integrative framework that brings together mechanism and trajectory, linking system-level influences with the inner work through which individuals interpret experiences, reconcile competing beliefs, and develop stable professional identities. Rather than replacing existing models of social learning or participation, the theory extends them by making explicit how professional identities are shaped, disrupted, and stabilized across the course of professional development.

This reframing has implications for how medical educators understand identity formation, design learning environments, and support learners through periods of uncertainty and transition. More broadly, it provides a conceptual foundation for future empirical inquiry into the processes through which professionals become who they are in practice. By making explicit the internal processes through which professional identities form, stabilize, and adapt, the KToS offers both a conceptual foundation for future inquiry and a practical lens for those who design, deliver, and support professional education across the health professions.

## Supplementary Information

Supplementary Material 1. Additional file 1. Features of a Complex Adaptive System (CAS)

Supplementary Material 2. Additional file 2. PRISMA-ScR Checklist.

Supplementary Material 3. Additional file 3. Search Strategy.

Supplementary Material 4. Additional file 4. Tabulated Summaries of Included Articles.

Supplementary Material 5. Additional file 5. AI-assisted Summaries of Included Articles.

## Data Availability

The datasets supporting the conclusions of this article are included within the article and its additional files.

## Abbreviations

SSR: Systematic Scoping Review
PIF: Professional Identity Formation
CAS: Complex Adaptive System
mSEBA: Modified Systematic Evidence-Based Approach
PCC: Population, Concept and Context
