# Supplementary material for: Professional socialization as a complex adaptive process: a systematic scoping review and integrative theory of professional identity formation
Source: BMC Med Educ. 2026 Jul 10;26:1582. doi: 10.1186/s12909-026-09894-1 (PMC13640327; doi:10.1186/s12909-026-09894-1)
Supplement: Supplementary file 1 [file 12909_2026_9894_MOESM1_ESM.docx]

**Additional File 1**. Characteristics of a complex adaptive system (CAS)

| Core CAS Elements | Features |
| --- | --- |
| Agent | A system element or part capable of responding to other agent actions and information   - Responses may include learning and adaptation. - The element may be an individual or collective process |
| Multiple agents with schemata | Informal, collaborative networks of individuals that partner and contribute to solution-making  Connectivity and interdependence between agents  Degrees of connectivity |
| Self-organising networks | Holistic patterns formed through human interactions  Causation  Feedback  The spontaneous emergence of new relationships, forms, or patterns of behaviour arising from repeated agent interactions over time |
| Co-evolution | An ongoing process in which agents are influenced by, and mutually adapt to, changes generated by agent interaction  Innovative pathways of governance emerge—a variety of what is known as “emergent behaviour” in CAS |
| System adaptation | The capacity of the system to adjust, evolve and respond to internal and external changes over time |
| Non-linearity | A characteristic of agent interaction generated by unpredictable agent responses to the actions of others |
| Feedback loops | Recursive mechanisms arising from multiple agent interactions over time that either reinforce (positive) or undermine (negative) each other.  Positive feedback loops support a change trajectory while negative feedback loops tend to undermine or negate change |
| Emergence | Complex patterns, behaviors, or properties arise from the interactions between the individual components of the system, rather than being predetermined or directly controlled by those components. |
| Boundaries | An artificial frame or socially constructed reference point which connects (not separates) a system with its environment  System fluidity means boundaries cannot be defined objectively |
| Far-from-equilibirum | A dynamic state in which complex systems maintain a stable appearance by balancing multiple interactions between diverse agents and feedback loops. Stability can be disproportionately disrupted by small changes |
| Path dependency | The influence of system history on current system behavior and events |
