# Supplementary material for: Professional socialization as a complex adaptive process: a systematic scoping review and integrative theory of professional identity formation
Source: BMC Med Educ. 2026 Jul 10;26:1582. doi: 10.1186/s12909-026-09894-1 (PMC13640327; doi:10.1186/s12909-026-09894-1)
Supplement: Supplementary file 3 [file 12909_2026_9894_MOESM3_ESM.docx]

**Additional File 2. Search Strategy**

| **Database** | **Date Searched** | **Search Input** |
| --- | --- | --- |
| **Scopus** | 21/06/24 | TITLE-ABS-KEY ( ( professional W/15 ( socialisation OR socialization OR "enculturation" OR "assimilation" OR "naturalisation" OR "acculturation" OR “naturalization” ) ) AND ( "professional identity" OR "professional identities" OR "professionalisation" OR "professionalization" OR "professionalism" OR "professional roles" OR "professional role" OR "professional behaviour" OR "professional behavior" ) ) AND PUBYEAR > 1999 AND PUBYEAR < 2025 |
| **ProQuest Education Database** | 23/06/24 | (noft("socialisation") OR "socialization" OR "enculturation" OR "assimilation" OR "naturalisation" OR "acculturation" OR "naturalization" OR MAINSUBJECT.EXACT("Socialization") AND noft("professional”)) AND (noft("professional identity" OR "professional identities" OR "professionalisation" OR "professionalization" OR "professionalism" OR "professional roles" OR "professional role" OR "professional behaviour" OR "professional behavior") AND MAINSUBJECT.EXACT("Professional identity")) [AND pd(20000101-20240531)](https://www.proquest.com/myresearch/savedsearches.checkdbssearchlink:rerunsearch/2648345/SavedSearches/$N?site=socscijournals&t:ac=SavedSearches) |
| **ProQuest Social Sciences Database** | 23/06/24 | oft(("professional") AND ("socialisation" OR "socialization" OR "enculturation" OR "assimilation" OR "naturalisation" OR "acculturation" OR "naturalization")) AND (("professional identity" OR "professional identities" OR "professionalisation" OR "professionalization" OR "professionalism" OR "professional roles" OR "professional role" OR "professional behaviour" OR "professional behavior") OR MAINSUBJECT.EXACT("Professional ethics") OR MAINSUBJECT.EXACT("Professional practice") OR MAINSUBJECT.EXACT("Professional development") OR MAINSUBJECT.EXACT("Professional identity")) AND pd(20000101-20240531) |
| **ProQuest Sociology Database** | 23/06/24 | [noft("professional identity" OR "professional identities" OR "professionalisation" OR "professionalization" OR "professionalism" OR "professional roles" OR "professional role" OR "professional behaviour" OR "professional behavior") AND (noft("socialisation" OR "socialization" OR "enculturation" OR "assimilation" OR "naturalization" OR "acculturation" OR "naturalisation") OR MAINSUBJECT.EXACT("socialisation")) AND pd(20000101-20240531)](https://www.proquest.com/myresearch/savedsearches.checkdbssearchlink:rerunsearch/2648351/SavedSearches/$N?site=sociology&t:ac=SavedSearches) |
| **PubMed** | 23/06/24 | (("professional socialisation"[Title/Abstract:~15] OR "professional socialization"[Title/Abstract:~15] OR "professional enculturation"[Title/Abstract:~15] OR "professional assimilation"[Title/Abstract:~15] OR "professional naturalisation"[Title/Abstract:~15] OR "professional naturalization"[Title/Abstract:~15] OR "professional acculturation"[Title/Abstract:~15] OR ("professional"[All Fields] AND ("socialization"[MeSH Terms] OR "acculturation"[MeSH Terms]))) AND ("professional identity"[Title/Abstract] OR "professional identities"[Title/Abstract] OR "professionalisation"[Title/Abstract] OR "professionalization"[Title/Abstract] OR "professionalism"[Title/Abstract] OR "professional roles"[Title/Abstract] OR "professional role"[Title/Abstract] OR "professional behaviour"[Title/Abstract] OR "professional behavior"[Title/Abstract] OR "social identification"[MeSH Terms] OR "professional role"[MeSH Terms])) AND (2000/1/1:2024/5/31[pdat]) |
| **ERIC** **(social sciences premium collection)** | 23/06/24 | [((((MAINSUBJECT.EXACT("Professionalism")) OR "professional") AND ("socialisation" OR "socialization" OR "enculturation" OR "assimilation" OR "naturalisation" OR "acculturalisation" OR "naturalization" OR MAINSUBJECT.EXACT("Socialization"))) AND ("professional identity" OR "professional identities" OR "professionalisation" OR "professionalization" OR "professionalism" OR "professional roles" OR "professional role" OR "professional behaviour" OR "professional behavior" OR MAINSUBJECT.EXACT("Professional Identity"))) AND pd(20000101-20240531)](https://www.proquest.com/myresearch/savedsearches.checkdbssearchlink:rerunsearch/2648359/SavedSearches/$N?site=eric&t:ac=SavedSearches) |
| **CINAHL** | 23/06/24 | TI ((professional AND (socialisation OR socialization OR enculturation OR assimilation OR naturalisation OR naturalization OR acculturation)) AND ("professional identity" OR "professional identities" OR professionalisation OR professionalization OR professionalism OR "professional roles" OR "professional role" OR "professional behaviour" OR "professional behavior")) OR AB ((professional AND (socialisation OR socialization OR enculturation OR assimilation OR naturalisation OR naturalization OR acculturation)) AND ("professional identity" OR "professional identities" OR professionalisation OR professionalization OR professionalism OR "professional roles" OR "professional role" OR "professional behaviour" OR "professional behavior"))  Publication Date: 20000101-20240531 on 2024-06-23 03:52 AM |
| **PsycINFO** | 23/06/24 | ((professional adj15 (socialisation or socialization or enculturation or assimilation or naturalisation or acculturisation or naturalization)) and ('professional identity' or 'professional identities' or 'professionalisation' or 'professionalization' or 'professionalism' or 'professional roles' or 'professional role' or 'professional behaviour' or 'professional behavior').ti,ab) |
| **EMBASE** | 23/06/24 | ((**professional** NEAR/15 (**socialisation** OR **socialization** OR **enculturation** OR **assimilation** OR **naturalisation** OR **acculturisation** OR **naturalization**)):ti,ab,kw) AND (**'professional identity'**:ti,ab,kw OR **'professional identities'**:ti,ab,kw OR **'professionalisation'**:ti,ab,kw OR **'professionalization'**:ti,ab,kw OR **'professionalism'**:ti,ab,kw OR **'professional roles'**:ti,ab,kw OR **'professional role'**:ti,ab,kw OR **'professional behaviour'**:ti,ab,kw OR **'professional behavior'**:ti,ab,kw) AND [01-01-2000]/sd NOT [01-06-2024]/sd |
| **ChatGPT5pro** | 23/09/25 | Prompt: what is known of the socialisation theory in medicine published in english between 1st january 2000 and 31st July 2025. pls create a list of papers (APA reference style) of ALL available and relevant papers |
| **Manus AI** | 23/09/25 | Prompt: what is known of the socialisation theory in medicine published in english between 1st january 2000 and 31st July 2025. pls create a list of papers (APA reference style) of ALL available and relevant papers |
