# Supplementary material for: Professional socialization as a complex adaptive process: a systematic scoping review and integrative theory of professional identity formation
Source: BMC Med Educ. 2026 Jul 10;26:1582. doi: 10.1186/s12909-026-09894-1 (PMC13640327; doi:10.1186/s12909-026-09894-1)
Supplement: Supplementary file 4 [file 12909_2026_9894_MOESM4_ESM.docx]

**Additional File 4.** Tabulated Summaries of Included Articles

| **S/N** | **Author, Year** | **Title** | **Type of Study** | **Introduction** | **Methods** | **Results** | **Conclusion** |
| --- | --- | --- | --- | --- | --- | --- | --- |
| 1 | Afshar et al., 2021 | Professional identity of medical students: Proposing a Meta Static Structural Model | Qualitative | The realization of professionalism and its desirable consequences, as the most important goal of medical education, primarily depends on identifying the process and mechanism of the professional identity formation, which in turn requires the accurate identification of components and structure of the concept of professional identity. Therefore, the aim of this study was to synthesize a static structural model for professional identity, based on the results of our previous research. | In this study the theory or model construction methodology was used to synthesize a static structural model of professional identity formation for medical students. In this regard, the Walker and Avant method was followed through three steps: specifying focal concepts, reviewing the literature, and organizing concepts into an integrated and efficient representation. | Based on the analysis of 9 selected conceptual models in the field of socialization and professional identity, first the key concepts of each model were extracted and then by carefully examining these concepts and determining their relationships and reviewing related texts, dimensions and components of professional identity were determined and presented in the form of a comprehensive structural static model. | The advantage of the proposed model over the existing models is the explicit presentation of the dimensions, constructs, and sub-constructs of the concept of professional identity. In addition, this model can be used as a general pattern in all non-medical professions. |
| 2 | Ahmadi et al., 2013 | Identity Formation of TEFL Graduate Students through Oral Discourse Socialization | Qualitative | This paper explores how the professional identity of the in-service teachers is affected by the socialization process, their prior histories and contextual factors. | It reports on academic discourse socialization of several EFL teachers in a TEFL graduate program in Iran through oral discourse practices over the first year of the program. | The epistemic stance of the student teachers in their preliminary interviews as well as in their small-group discussions showed how they lived up to the self-images as EFL teachers they created mainly based on their personal histories. The interviews with the participants also showed that teachers‟ professional identity construction was influenced more by the personal histories and prior beliefs concerning teaching and learning a foreign language than accumulating pedagogical and theoretical knowledge in pre-service teacher education courses or through traditional professional development programs. Contextual factors prevent the student teachers from adopting and embracing pedagogical practices in harmony with their conceptual images of modern ways of learning and teaching a foreign language. | It concludes that dialogical interactions through oral discourse practices are likely to result in re-consideration of their prior professional identity. The paper is likely to make a contribution to our understanding of professional identity construction of EFL teachers in graduate programs as far as academic oral discourse practices are concerned. |
| 3 | Al-Omary et al., 2024 | Implementing learning into practice from continuous professional development activities: a scoping review of health professionals’ views and experiences | Review | Continuing professional development (CPD) is an approach for health professionals to preserve and expand their knowledge, skills, and performance, and can contribute to improving delivery of care. However, evidence indicates that simply delivering CPD activities to health professionals does not lead to a change in practice. This review aimed to collate, summarize, and categorize the literature that reported the views and experiences of health professionals on implementing into practice their learning from CPD activities. | This review was guided by the Joanna Briggs Institute Reviewers’ Manual methodology for scoping reviews. Three databases, PubMed, Embase and Cumulative Index to Nursing and Allied Health Literature (CINAHL), were systematically searched in February 2023 for articles published since inception. Two independent reviewers screened the articles against the inclusion criteria, and completed the data extraction. Data were summarized quantitatively, and the findings relating to views and experiences were categorized into challenges and facilitators. | Thirteen articles were included. Implementation of learning was not the primary focus in the majority of studies. Studies were published between 2008-2022; the majority were conducted in North America and nurses were the most common stakeholder group among Healthcare Professionals (HCPs). Five studies adopted qualitative methods, four quantitative studies, and four mixed-methods studies. The reported barriers of implementation included lack of time and human resource; the facilitators included the nature of the training, course content and opportunity for communal learning. | This review highlights a gap in the literature. Available studies indicate some barriers for health professionals to implement their learning from CPD activities into their practice. Further studies, underpinned with appropriate theory and including all relevant stakeholders are required to investigate strategies that may facilitate the integration of learning from CPD into routine practice. |
| 4 | Alharbi et al., 2020 | Undergraduate nursing students' adoption of the professional identity of nursing through social media use: A qualitative descriptive study | Qualitative | Previous research has addressed nursing course components such as clinical placements that affect students' socialisation processes and the development of their professional identities. Social media is an increasingly important factor that has not yet been explored in-depth in terms of its influence on the development of the professional identity of nursing and is a major gap in our knowledge and understanding of undergraduate nurses' development. | Semi-structured and audio-recorded interviews were undertaken between November 2018 and January 2019. The transcribed data were thematically analysed using NVivo 12. | Three main themes emerged: Learning about nursing professional identity through social media, adopting the identity of nursing, and students sharing their understanding of nursing professional identity via social media. Students used social media to learn about the nursing profession, and this helped them to develop a sense of belonging to the nursing profession. The students also used social media to share their experiences as nursing students and to enhance society's understanding of the importance of the nursing profession. | Undergraduate nursing students should be encouraged to develop their professional identity through socialisation. Social media can facilitate the socialisation process of undergraduate nursing students by connecting them with members of the nursing community. |
| 5 | Ardi et al., 2022 | Professional identity formation of medical students: A mixed‑methods study in a hierarchical and collectivist culture | Cross-sectional, Qualitative | Professional identity formation (PIF) has been recognized as an integral part of professional development in medical education. PIF is dynamic: it occurs longitudinally and requires immersion in the socialization process. Consequently, in the medical education context, it is vital to foster a nurturing learning environment that facilitates PIF. This study assesses PIF among medical students in various stages of study and explores their perceptions of PIF, with its contributing and inhibiting factors. | This mixed-methods study uses a sequential explanatory approach with undergraduate (years 2, 4, and 6) and postgraduate medical students in Indonesia. We examine the subjects by administering an adapted questionnaire on PIF. We completed a series of FGDs following questionnaire administration. Quantitative and thematic analyses were conducted sequentially. A total of 433 respondents completed the questionnaire. | There were statistically significant differences among subjects on the subscales “Recognition and internalization of professional roles” and “Self-control in professional behaviour”; the more senior students had higher scores. We conducted 6 FGDs in total. The results characterize PIF as a complex, dynamic, and longitudinal journey to becoming a medical doctor that is closely related to a student’s motivation. The FGDs also highlight the importance of both internal factors (students’ values, attributes, and personal circumstances) and external factors (curriculum, the learning environment, workplace-based learning, and external expectations) for PIF in medical education. | Higher-level students show higher scores in some aspects of PIF, which further validates the potential use of the questionnaire to monitor PIF, a dynamic process influenced by internal and external factors. Generating awareness among medical students and encouraging reflection on their PIF stage may be crucial for PIF processes. |
| 6 | Bandura, 2004 | Health Promotion by Social Cognitive Means | Review | This article examines health promotion and disease prevention from the perspective of social cognitive theory. This theory posits a multifaceted causal structure in which self-efficacy beliefs operate together with goals, outcome expectations, and perceived environmental impediments and facilitators in the regulation of human motivation, behaviour, and well-being. |  | Belief in one’s efficacy to exercise control is a common pathway through which psychosocial influences affect health functioning. This core belief affects each of the basic processes of personal change—whether people even consider changing their health habits, whether they mobilize the motivation and perseverance needed to succeed should they do so, their ability to recover from setbacks and relapses, and how well they maintain the habit changes they have achieved. | Human health is a social matter, not just an individual one. A comprehensive approach to health promotion also requires changing the practices of social systems that have widespread effects on human health. |
| 7 | Bremer et al., 2022 | How an EPA-based curriculum supports professional identity formation | Qualitative | Entrustable professional activities (EPAs) are widely used in medical education, and they might be an important incentive to stimulate professional identity formation (PIF) of medical students, by actively encouraging participation in the workplace. The goal of this study was to explore the effects of an EPA-based curriculum on the PIF of medical students in undergraduate curricula. | In this study at the Radboud University Medical Center in Nijmegen, the Netherlands, the authors interviewed twenty-one medical students in three focus group interviews (November 2019), and conducted a thematic analysis based on both the synthesizing concepts PIF, communities of practice and EPAs, and newly defined themes. | Four central themes proved crucial for understanding the influence of EPAs on PIF: creating learning opportunities, managing feedback, dealing with supervision in context and developing confidence. EPAs helped students to create learning opportunities and to choose activities purposefully, and the use of EPAs stimulated their feedback seeking behaviour. The context and way of supervision had a great impact on their development, where some contexts offer better learning opportunities than others. EPAs helped them develop trust and self-confidence, but trust from supervisors hardly appears to result from using EPAs. | An EPA-based curriculum does stimulate PIF in the complex context of working and learning by supporting participation in the workplace and by encouraging feedback-seeking behaviour. Striking the right balance between participation, feedback-seeking behaviour and choosing learning activities is essential. |
| 8 | Chen et al., 2022 | Developing an innovative medical ethics and law curriculum—constructing a situation-based, interdisciplinary, court-based learning course: a mixed methods study | Qualitative | Traditional lecture‐based medical ethics and law courses deliver knowledge but may not improve students’ learning motivation. To bridge this theory‐to‐practice gap and facilitate students’ learning effectiveness, we applied situated‐learning theory to design an interdisciplinary court‐based learning (CBL) component within the curriculum. Our study aimed to investigate students’ learning feedbacks and propose a creative course design. | A total of 135 fourth‐year medical students participated in this course. The CBL component included 1 h of introduction, 1 h of court attendance, and 2 h of interdisciplinary discussion with senior physicians, judges, and prosecutors. After the class, we conducted a survey using a mixed‐methods approach to gauge students’ perceptions of engagement, performance, and satisfaction. | A total of 97 questionnaires were received (72% response rate). Over 70% of respondents were satisfied and felt that the class was useful except for role‐playing activities (60%). More than 60% reported a better understanding of the practical applications of medical law. Approximately half (54%) reported less anxiety about medical disputes. 73% reported that the lecture provided awareness of potential medical disputes, and most respondents expressed an interest in medical law courses after the court visit (78%). 80% of the respondents were able to display empathy and apply mediation skills. Qualitative analyses showed that students demonstrated new knowledge, including recognizing the significance of the medical profession, distinguishing the importance of physician‐patient communication, having confidence in the fairness of the justice system, and being willing to increase their legal knowledge. | CBL curriculum increases students’ learning motivation in strengthening medical professionalism and medical law, develops students’ empathy for patients and communication skills, as well as builds up students’ trust in the justice system. This novel course design can be applied to teach medical ethics and law. |
| 9 | Cho et al., 2024 | Becoming a doctor: using social constructivism and situated learning to understand the clinical clerkship experiences of undergraduate medical students | Qualitative | Despite the emphasis on the uniqueness and educational importance of clinical clerkships in medical education, there is a lack of deep understanding of their educational process and outcomes. Especially due to an inherent trait of clinical clerkships which requires participation in the workplace outside the classroom, it is difficult to fully comprehend their educational potential using traditional learning perspectives such as imbibing outside knowledge. Accordingly, this study aims to explore the experiences of a rotation-based clerkship of medical school students from the perspective of social constructivism of learning, which can empirically examine what and how  medical students learn during clinical clerkship in South Korea. By providing an insight into the workings of the  clerkship process, this study contributes to a better understanding of how a learning-friendly environment can be cultivated at clinical clerkships. | The study utilized a basic qualitative study to understand what and how medical students learn during  their clinical clerkships. Semi-structured, in-depth individual interviews were conducted with eight sixth-graders who had experienced a two-year clerkship at Ajou University Medical School. Data were analyzed based on Lave and Wenger’s situated learning theory and Wenger’s social theory in learning. | We found that the medical students had developed different aspects of their professional identities such  as values, functionality, career decisions, sociality, and situating during their clinical clerkships. Further, professional identity was formed through a combination of participation and reification—the processes involved in the negotiation of meaning. This combination was facilitated by the students’ first experience and relationships with professors, classmates, and patients. Finally, non-learning occurred in the context of over-participation (learning anxiety and alienation) or over-reification (evaluation and e-portfolio). | This study revealed five sub-professional identities and their formation process from the learners’  perspective, thereby uncovering the unique learning characteristics and advantages of rotated-based clerkship and contributing to a further understanding of how gradual improvements can be made to the traditional clerkship education of medical students. |
| 10 | Cox and Simpson, 2020 | Cultural Humility: A Proposed Model for a Continuing Professional Development Program | Review | Continuing professional development (CPD) is an essential component of professional practice for registered health practitioners to maintain and enhance knowledge, skills and abilities. There are many topics that practitioners may pursue relevant to their practice environment, and, in recent years, providing culturally safe and respectful practice is an emerging area of need. Unfortunately, many health professionals, whilst willing to offer cultural safe healthcare, may be uncertain of how to enact that practice. The World Health Organisation recognises attainment of the highest possible standard of health as a basic human right, and cultural safety is increasingly becoming an expectation of health professionals. | To address this need and the insufficiency of support in the literature, the authors have presented a discussion paper on various aspects of cultural safety and the underlying constructs, such as cultures, that support it. The discussion takes into account core constructs that signpost the path to cultural safety and recognises the role and accountability of all levels of the healthcare system, not merely the practitioner. | The authors propose a model program for a cultural humility CPD activity incorporating pre-work, online modules, interactive workshop, reflection on professional practice and a post-workshop evaluation. | Cultural humility is an active and ongoing process that focusses on self-reflection and facilitates the client becoming the teacher and co-creating their health experience and health outcomes. It encourages interactions that are respectful of cultural beliefs, and, thus, we believe that it is a necessary component of all culturally safe healthcare provision. It therefore needs to be included in the ongoing CPD of health practitioners. Culturally safe healthcare, however, also requires input of community members. Thus, any educational intervention would require collaboration and participation from patients and community leaders from all cultural groups. An interactive program such as the one discussed in this paper could enhance the provision of culturally safe care and meet CPD requirements for practising and intern pharmacists. |
| 11 | Davitadze et al., 2022 | SIMBA: using Kolb's learning theory in simulation-based learning to improve participants' confidence | Interventional | Simulation via Instant Messaging- Birmingham Advance (SIMBA) delivers simulation-based learning (SBL) through WhatsApp and Zoom based on Kolb’s experiential learning theory. This study describes how Kolb’s theory was implemented in practice during SIMBA adrenal session. | SIMBA adrenal session was conducted for healthcare professionals and replicated Kolb’s 4-stage cycle: (a) concrete experience—online simulation of real-life clinical scenarios, (b) reflective observation—discussion and Q&A following simulation, (c) abstract conceptualisation—post-session MCQs, and (d) active experimentation—intentions to implement the acquired knowledge in future practice. Participants’ self-reported confidence levels for simulated and non-simulated cases pre- and post-SIMBA were analysed using Wilcoxon Signed-Rank test. Key takeaway and feedback were assessed quantitatively and qualitatively in a thematic analysis. | Thirty-three participants were included in the analysis. A Wilcoxon signed-rank test showed that the SIMBA session elicited a statistically significant change in participants’ self-reported confidence in their approach to Cushing’s syndrome (Z = 3.873, *p* = 0.0001) and adrenocortical carcinoma (Z = 3.970, *p* < 0.0001). 93.9% (*n* = 31/33) and 84.8% (*n* = 28/33) strongly agreed/agreed the topics were applicable to their clinical practice and accommodated their personal learning style, respectively. 81.8% (*n* = 27/33) reported increase in knowledge on patient management, and 75.8% (*n* = 25/33) anticipated implementing learning points in their practice. | SIMBA effectively adopts Kolb’s theory to provide best possible experience to learners, highlighting the advantages of utilising social media platforms for SBL in medical education. The ability to conduct SIMBA sessions at modest cost internationally paves way to engage more healthcare professionals worldwide. |
| 12 | Engeström, 2021 | Expansive Learning at Work: Toward an activity theoretical reconceptualization | Review | Cultural-historical activity theory has evolved through three generations of research. The emerging third generation of activity theory takes two interacting activity systems as its minimal unit of analysis, inviting us to focus research efforts on the challenges and possibilities of inter-organizational learning. | Activity theory and its concept of expansive learning are examined with the help of four questions: 1. Who are the subjects of learning? 2. Why do they learn? 3. What do they learn? 4. How do they learn? | Five central principles of activity theory are presented, namely activity system as unit of analysis, multi-voicedness of activity, historicity of activity, contradictions as driving force of change in activity, and expansive cycles as possible form of transformation in activity. Together the four questions and five principles form a matrix which is used to present a study of expansive learning in a hospital setting in Finland. In conclusion, implications of the framework for the understanding of the increasingly important horizontal dimension of learning are discussed. | People habitually tend to depict learning and development as vertical processes, aimed at elevating humans upward, to higher levels of competence. Rather than simply denounce this view as an outdated relic of enlightenment, the author suggests the construction of a complementary perspective, namely that of horizontal or sideways learning and development. The case discussed in this paper provides rich indications of such a complementary dimension. |
| 13 | Hafferty, 2008 | Professionalism and the Socialization of Medical Students | Review | In this chapter, the author will examine issues of professions and professionalism through a particular lens, socialization theory. The fundamental assumption driving this chapter is the author’s belief that current discussions about professionalism contain a bevy of unexamined assumptions about what happens to trainees as they move from the social and social-psychological status of lay outsiders to full members in a particular occupational group, in this case, medicine. These tacit renderings, in turn, block or otherwise distort meaningful efforts by medical educators to link medical training with principles and practices of professionalism. Until these disconnects and contradictions are pinpointed and made more explicit, efforts to develop targeted and effective educational interventions will continue to be thwarted. | The author organizes this chapter in four parts. First, a brief overview of the modern-day professionalism movement and its emergent discourse of nostalgic professionalism is presented. This discourse is then subjected to a critical review by highlighting its tacit assumptions and conceptual weaknesses. Second, and taking an ‘‘if-then’’ form of argument, it is concluded that if professionalism is to be conceptualized as values, then this calls for a particular framing of medical education – in this case education-as-socialization. To this end, the author briefly reviews some principles of socialization and apply them to the particular case of medical education as professional preparation. Third, a more explicit look at professionalization as a form of social control and the implications of such a framing for a socialization approach to medical education is taken. The author closes with some thoughts on how better to frame medical education as socialization. |  | It remains vitally important that we ponder about the purpose of medical education - whether it is considered to be the impartation of basic knowledge, skills, and requisite behaviors, or also the inclusion of concepts such as beliefs, normative behaviors, and even values. In many respects, the choice remains medicine’s given its continued control over the selection and training of future physicians. Finally, there is the issue of authenticity, if professionalism is referred to as the product of deep or surface learning. Medicine trains for the usual and commonplace as well as the unusual and idiosyncratic. It is relatively easy to be professional, and even easier to appear so, within the usualness of everyday medical life. At the same time, every work setting has the unanticipated and often unanticipatable events that truly stretch the boundaries of an occupation’s knowledge, skills, and values. This is why training in professionalism functions – and should function – at the level of socialization and at the level of values and identity. Case studies are important pedagogical tools. They help students to grasp particulars. So too is the situated learning that takes place on the job as individuals ‘‘learn the ropes’’ and ‘‘the way things are done around here.’’ |
| 14 | Harman et al. 2021 | Physiotherapy: an active, transformational, and authentic career choice | Qualitative | To explore the process of physiotherapy student professional identity development and the evolution of expectations and views of interprofessional practice in the first year of their program. | One-on-one interviews at three time points: after being accepted into the physiotherapy program and before classes began (T1; n = 12); after term one (T2; n=9) and on completion of year one (T3; n = 7). Analyses employed narrative methodology, guided by anticipatory socialization and professional identity theories. | At T1, participants described their path towards physiotherapy, indicating career satisfaction as the core of their choice. Expectations of practice aligned with the normative social positioning of the profession. T2 and T3 interviews revealed that their pre-entry conceptualization of practice was both challenged and positively reinforced, leading them either to being satisfied with, or questioning their choice. Clinical placements created the most meaningful opportunities to understand their roles both as individual professionals and members of a collaborative team. | Findings revealed the complex process of professional socialization in physiotherapy students with implications for admissions and formal and IPE curricula. |
| 15 | Haslam et al., 2009 | Social Identity, Health and Well-Being: An Emerging Agenda for Applied Psychology | Review | The social environment comprising communities, families, neighbourhoods, work teams, and various other forms of social group is not simply an external feature of the world that provides a context for individual behaviour. Instead these groups impact on the psychology of individuals through their capacity to be internalised as part of a person’s social identity. If groups provide individuals with a sense of meaning, purpose, and belonging (i.e. a positive sense of social identity) they tend to have positive psychological consequences. | The impact of these identity processes on health and well-being is explored in the contributions to this special issue.  In this editorial, the authors discuss these contributions in light of five central themes that have emerged from research to date. These themes address the relationship between social identity and (a) symptom appraisal and response, (b) health-related norms and behaviour, (c) social support, (d) coping, and (e) clinical outcomes. |  | The special issue as a whole points to the capacity for a social identity approach to enrich academic understanding in these areas and to play a key role in shaping health-related policy and practice. |
| 16 | Horsbough and Ippolito, 2018 | A skill to be worked at: using social learning theory to explore the process of learning from role models in clinical settings | Qualitative | Role modelling is widely accepted as being a highly influential teaching and learning method in medical education but little attention is given to understanding how students learn from role models. This study focuses on role modelling as an active, dynamic process, involving observational learning and aims to explore the process involved, including strategies that learners and medical teachers use to support this. | To gain insight into medical students’ and clinical teachers’ understanding of learning through role modelling, a qualitative, interpretative methodology was adopted, using one-to-one semi-structured interviews. Six final year medical students and five clinical teachers were purposefully sampled and interviewed. Interviews were audio recorded and transcribed. The data were then analysed using open and axial coding before codes were combined to develop broader themes. | Students could identify ways in which they learnt from role models but acknowledged that this was complex and haphazard. They described selectively and consciously paying attention, using retention strategies, reproducing observed behaviour and being motivated to imitate. Students evidenced the powerful impact of direct and vicarious reinforcement. Clinical teachers reported using strategies to help students learn, but these were not always consciously or consistently applied or informed by teachers’ understanding of their students’ cognitive processing. | Findings illustrate in what ways the process of learning from role models in clinical settings is challenging. They also support the relevancy and usefulness of Bandura’s four stage social learning model for understanding this process and informing recommendations to make learning from role modelling more systematic and effective. |
| 17 | Hoydalsvik, 2019 | The hidden professionals? An interview study of higher education-based teacher educators ́professional identity | Qualitative | This article investigates teacher educators’ self-understanding by asking how they explain their professional identities as teacher educators, based on socialisation and further professional development. Teacher educators facilitate learning from the initial teacher education phase to in-service teachers’ further professional development. | The data consists of thirteen qualitative semi-structured interviews and two focus-group interviews with teacher educators from two universities in Norway. | Using Bryman’s four-stage approach of analysis, 15 categories were re-organised into three main categories: (1) Recruitment and socialisation, (2) Professional identity, and (3) Professional development. | The findings illustrate that teacher educators have different understandings of being a professional. For some, their identity is rooted in the discipline in which they were educated. However, others have built identities as teacher educators supplementary to their primary careers. This knowledge has implications for how universities can and should support teacher educators in the development of their identities. This can apply to educational strategies, institutional leadership and the role of teacher educators themselves. Their further professional development can be facilitated by a clearer understanding and appreciation of this profession reflected in educational policy. |
| 18 | Hunter et al., 2007 | Becoming a Scientist: The Role of Undergraduate Research in Students’ Cognitive, Personal, and Professional Development | Qualitative | Ongoing and current debates in the academic literature concerning how learning occurs, how students develop intellectually and personally during their college years, and how communities of practice encourage these types of growth posit effective practices and the processes of students’ cognitive, epistemological, and interpersonal and intrapersonal development. Although a variety of theoretical papers and research studies exploring these topics are widely published, none has yet focused on intensive, summer apprentice-style UR experiences as a model to investigate the validity of these debates. | In this ethnographic study of summer undergraduate research (UR) experiences at four liberal arts colleges, where faculty and students work collaboratively on a project of mutual interest in an apprenticeship of authentic science research work, analysis of the accounts of faculty and student participants yields comparative insights into the structural elements of this form of UR program and its benefits for students. | Comparison of the perspectives of faculty and their students revealed considerable agreement on the nature, range, and extent of students’ UR gains. Specific student gains relating to the process of “becoming a scientist” were described and illustrated by both groups. Faculty framed these gains as part of professional socialization into the sciences. In contrast, students emphasized their personal and intellectual development, with little awareness of their socialization into professional practice. | Viewing study findings through the lens of social constructivist learning theories demonstrates that the characteristics of these UR programs, how faculty practice UR in these colleges, and students’ outcomes—including cognitive and personal growth and the development of a professional identity—strongly exemplify many facets of these theories, particularly, student-centered and situated learning as part of cognitive apprenticeship in a community of practice. |
| 19 | Isba et al., 2017 | Social network analysis in medical education | Review | Humans are fundamentally social beings. The social systems within which we live our lives (families, schools, workplaces, professions, friendship groups) have a significant influence on our health, success and well-being. These groups can be characterised as networks and analysed using social network analysis. | Social network analysis is a mainly quantitative method for analysing how relationships between individuals form and affect those individuals, but also how individual relationships build up into wider social structures that influence outcomes at a group level. Recent increases in computational power have increased the accessibility of social network analysis methods for application to medical education research. | Social network analysis has been used to explore team-working, social influences on attitudes and behaviours, the influence of social position on individual success, and the relationship between social cohesion and power. This makes social network analysis theories and methods relevant to understanding the social processes underlying academic performance, workplace learning and policy-making and implementation in medical education contexts. | Social network analysis is underused in medical education, yet it is a method that could yield significant insights that would improve experiences and outcomes for medical trainees and educators, and ultimately for patients. |
| 20 | Janke et al., 2021 | A Pathway to Professional Identity Formation: Report of the 2020-2021 AACP Student Affairs Standing Committee | Qualitative | Professional identity formation (PIF) involves internalizing and demonstrating the behavioural norms, standards, and values of a professional community, such that one comes to “think, act and feel” like a member of that community. Professional identity influences how a professional perceives, explains, presents and conducts themselves. | This report of the 2020-2021 AACP Stu- dent Affairs Standing Committee (SAC) describes the benefits of a strong professional identity, including its importance in advancing practice transformation. This report responds to a recommendation from the 2019-2020 SAC, | This report presents an illustrative and interpretative schema as an initial step towards describing a pharmacist’s identity. However, the profession must further elucidate a universal and distinctive pharmacist identity, in order to better support pharmacists and learners in explaining and presenting the pharmacist’s scope of practice and opportunities for practice change. Additionally, the report outlines recommendations for integrating intentional professional identity formation within professional curricula at colleges and schools of pharmacy. | Although there is no standardized, single way to facilitate PIF in students, the report explores possibilities for meeting the student support and faculty development needs of an emerging new emphasis on PIF within the Academy. |
| 21 | Johnson et al., 2023 | Preparing Pharmacy Educators as Expedition Guides to Support Professional Identity Formation in Pharmacy Education | Qualitative | To provide an educator-friendly travel guide for supporting pharmacy students’ lifelong journey to professional identity formation. | Existing literature on professional identity formation was analysed. | In contrast to professionalism, which has emphasized externally visible behaviours, professional identity focuses on the internalization of the attitudes, standards, and behavioural norms of a profession, such that one “thinks, acts, and feels” like a member of that profession. Identity, whether personal or professional, is continuously developed in part during interactions with others and in response to internal and external feedback on those interactions. Educators play a critical role in helping students navigate the “provocative moments” (e.g., transitions, dissonance) that accompany identity formation. To help educators travel with purpose, several identity formation theories suggest means of creating learning experiences and supporting the development of a professional identity. Additionally, guidebooks for the trip (i.e., published literature) provide examples of didactic and experiential teaching approaches that can be used to promote professional identity formation. While further exploration and research are necessary, traveling this journey with colleagues can help members of the Academy succeed in sustainably and effectively infusing intentional professional identity formation within pharmacy education and training. | There are myriad ways for educators to develop and support professional identity formation, which can present a challenge when defining the role that educators play in this complex, dynamic process. Educators must understand the reasoning behind various approaches and the common dialogue used to engage and support learners as their expedition guides on the lifelong journey to professional identity formation. |
| 22 | Kamel-ElSayed and Loftus, 2018 | Using and Combining Learning Theories in Medical Education | Qualitative | Medical education needs to be well informed by learning theories, and preferably more than one theory. Medical teachers need to be familiar with a range of learning theories and should be able to critique them in ways that allow us to combine good ideas from one theory with good ideas from other theories. This can give their teaching real depth and help avoid common pitfalls that arise from misapplying a theory due to a superficial understanding. | The authors provide an example where a number of learning theories (constructivism, threshold concepts, narrative inquiry, dialogism and active learning) were usefully combined. Students were supplied with copies of the thyroid gland physiology PowerPoint slides several days before the lecture and directed to come to class prepared to discuss the content. The lecture itself was delivered using an interactive, narrative approach. The interactions followed a storytelling format that incorporated simple questions and answers at regular intervals using a version of the PowerPoint slides prepared specifically for that purpose. Following the session, a preliminary study was conducted to evaluate the learners’ perceptions by using both questionnaire and focus groups. | Feedback was very positive with comments finding the session engaging compared to other lectures. Students found it easier to pay attention and follow due to the interactive atmosphere which promotes listening and thinking in a way they are not used to. It builds curiosity, making students question what other people are saying. | The main point the authors wish to make is that by aligning the different theoretical levels and combining insights from several educational theories, they were able to come up with a teaching/learning design that had real theoretical depth and allowed us to analyze what needed to happen, and what did happen, and how they can improve it in the future. In combining these different theories in a rigorous manner, the authors have come to a deeper understanding of what can be happening in the complexities of interactive teaching. All the theories mentioned provided them with insights into how they can make sense of classroom activity. The theories all align with each other at the different epistemological levels. This approach has provided them with a robust sense of direction, both in designing the learning activities, and in reflecting on what happened and how they can improve for the future. |
| 23 | Kao and Chen, 2017 | Am I a teacher? How educare givers in Taiwan construct their professional identity during socialisation into public preschools | Qualitative | A new job position, “educare giver,” which was created after kindergartens and preschools in Taiwan were integrated in 2012, has caused several problems such as ambiguity in the role of childcare givers and inequity in the division of labour. Studies have provided a limited scope and have not examined the identities and struggles of educare givers at the individual and organisational levels. This study investigated the effect of contextual factors on the professional identities of educare givers during their socialisation. | The researchers adopted a qualitative research design and conducted in-depth interviews with 20 educare givers at public preschools. | The findings reveal that educare giver identities are those as teachers, labourers, and teaching and administrative assistants. Contextual factors that affect the construction of their identities at the three stages of their socialisation are information channels, educational background, the recruitment pro- cess, titles, the attitudes of stakeholders, and job assignments. | The findings show that the professional identities of preschool educare givers are dynamic and reconstructed through various stages of their experiences. These factors influence the identity types of educare givers. In conclusion, the identity of educare givers is a product of continual negotiation and the reconstruction of power in multiple contexts. Their identity has multiple and fluid traits at various stages and in diverse contexts. |
| 24 | Kennie-Kaulbach et al., 2024 | A Narrative Inquiry of Critical Events Supporting Professional Identity Formation in Introductory Pharmacy Practice Experiences | Qualitative | Professional identity formation (PIF) is the process of internalizing a profession’s core values and beliefs so that one begins to think, act, and feel like a member of that profession. PIF occurs over an individual’s professional socialization in stages, precipitated by critical moments or experiences. The purpose of this study was to answer the following: (1) What are the critical events for pharmacy student PIF in introductory pharmacy practice experiences?; and (2) How do these events support or challenge PIF? | This study used critical event narrative inquiry methodology. A critical event is an experience that creates a change in understanding and affects professional performance. Semi structured interviews were conducted with pharmacy students who completed introductory pharmacy practice experience rotations in community (first year) and hospital (second year) settings. Interviews were coded and analysed using narrative analysis. | Twelve first-year and 10 second-year students participated in this study. Narratives revealed that common experiential education experiences resulted in a deeper understanding of core role elements (i.e., professional autonomy, responsibility, interprofessional collaboration, and patient-centered care), leading to changes in agency, knowledge, and anticipated future behaviours. While first-year participants’ narratives mainly focused on understanding pharmacist roles (“what”), second-year participants focused on understanding the process to fulfil those roles (“how to”). Emotions, assuming responsibility, external validation, and preceptor guidance supported these events. | Critical events in introductory pharmacy practice experiences affect and shape pharmacy students’ PIF. These events are relevant to pharmacy education because these experiences can result in changes in knowledge, agency, or future behaviours for students. |
| 25 | Kiran et al., 2023 | Transforming professional identity of medical teachers in Pakistan by a certificate program in health professions education: a thematic analysis of reflective essays | Qualitative | A teacher’s professional identity development is a dynamic and continuous process that requires rigorous, longitudinal, faculty development initiatives which are designed to work on individual professional growth. Impact of such programs must be evaluated by qualitative means to identify the process of change; The purpose of our study was to investigate whether and how our thoughtfully designed 6-month certificate program has transformed the professional identity of medical teachers. | The study was conducted in National University of Medical Sciences, Pakistan using thematic analysis, wherein a weekly task of guided reflective writing, on Moodle, was given. Data was analysed in six phases to achieve credibility and transferability. | By analysing 202 reflective writings, seven subthemes were identified which manifest transformation in certain aspects of identity of participants and grouped under three major themes. The seven subthemes represent transformative journey of participants and include recognizing millennial learners’ dilemma, identifying learning gaps and overcoming barriers, discovering a newer version of self, alternative frame of thinking, transforming traditional classroom, conducive learning environment and Community of Practice. Three major themes identified were Awareness, Modelling and Socialization which represent three processes bringing transformation in participants. | Our faculty development program has transformed certain aspects of professional identities of medical teachers by incorporating informal teaching strategies of experiential learning, professional socialization, reflections, and role modelling. Participants’ beliefs and practices on teaching were challenged by giving a disorienting dilemma of millennial learners and learning theories. They underwent critical discourse with professional peers and mentors in community of practice, reflected on their traditional teaching practices, acquired new insight, underwent self-discovery, and introduced digitalization and interactive learning strategies within their classrooms. |
| 26 | Lane et al., 2019 | A model of the factors influencing teaching identity among life sciences doctoral students | Qualitative | One barrier to the adoption of evidenced-based teaching practices may be that faculty do not see teaching as an important part of their identities as professionals. Graduate school is a key time for professional identity development, and currently, we know little about how doctoral students develop identities as college teachers. | In this qualitative study, we aim to characterize the factors that promote and hinder teaching identity among 33 life sciences doctoral students with diverse career interests at one research university. We collected data using semi-structured interviews and analysed it using qualitative analysis closely aligned with grounded theory. Our analysis involved iteratively and collaboratively analysing interview transcripts while considering existing literature about socialization and professional identity and remaining open to novel ideas in the data. | From this analysis, we developed a mechanistic model of the factors that influenced teaching identity in our participants. Independent teaching experiences, teaching professional development, and teaching mentors contributed to salient and stable teaching identities among doctoral students. Being recognised by faculty as a teacher was also important, but rare. The professional culture that doctoral students perceived acted like a blizzard that they had to navigate through to develop a teaching identity. This culture strongly valued research over teaching, resulting in a sometimes cold and isolating environment for students interested in teaching. The culture also made it harder to see existing opportunities for teaching development and made it more challenging to move toward these opportunities, much like the deep snow and driving winds of a blizzard. | The mechanistic model described in this work is an important first step in understanding how doctoral training influences teaching identity. This model serves as a hypothesis that should be tested and refined through additional empirical work across contexts. |
| 27 | Liu et al., 2024 | Public health education using social learning theory: a systematic scoping review | Review | Public health education (PHE) in social environments plays a crucial role in mitigating the impact of public health events, especially with the recent surge in global incidents. Social learning theory (SLT) provides a strong theoretical foundation for implementing PHE. The objective of this study is to conduct a systematic scoping review of PHE using SLT, synthesizing the target populations, types of research, main findings, and future directions. | The study followed the Preferred Reporting Items for Systematic Reviews and Meta‐Analysis Extension for Scoping Review (PRISMA‐ScR) guidelines. We conducted a comprehensive search of five electronic databases (Web of Science, Scopus, PubMed, ProQuest, and APA PsycInfo) for English articles related to PHE using SLT. Two reviewers independently screened the titles and abstracts. Descriptive statistics were utilized to analyze the characteristics of the articles included in the study, followed by a comprehensive narrative analysis of the results. | Research on PHE using SLT mainly focuses on adolescents, students, special patients, and vulnerable populations. The study sample includes seven research types and nine commonly used experimental methods. Four modes of PHE using SLT are identified, along with four types of summarized research results. | PHE research based on SLT can be prioritized for preventing widespread infectious diseases, spreading fundamental public health information, and assisting patients with particular illnesses. To enhance the implementation of PHE, researchers and policymakers should integrate online and offline health education resources, ensure the accessibility of up‐to‐date information, and leverage digital technologies in PHE. More highly interactive and participatory health education courses will be established in social learning environments to encourage public participation in PHE. |
| 28 | London et al., 2021 | The Pioneers’ Stories As A Tool For Introducing ­ Graduate Students To The Engineering Education Research ­ Community | Qualitative | This study, framed with a socialization lens, discusses the impact of using stories about early contributors in engineering education (via results of the Engineering Education Pioneers Project) as a mechanism to help socialize the first cohort of graduate students in a new engineering education Ph.D. program. | Two instructors designed a new course requiring five students to read selected pioneers’ stories weekly. Students subsequently wrote weekly reflections about the stories and submitted a final synthesis of their reflections at the end of the semester. | Findings presented using a student-led autoethnographic approach include highlights and themes describing the overall impact of the pioneers’ stories to facilitate an understanding of the field and develop an identity as an engineering education researcher. Findings also include the instructors’ observations of the stories’ impact on students’ professional formation. | Implications for various stakeholders in the engineering education ecosystem are discussed. |
| 29 | May and Finch, 2009 | Implementing, Embedding, and Integrating Practices: An Outline of Normalization Process Theory | Review | Understanding the processes by which practices become routinely embedded in everyday life is a long-standing concern of sociology and the other social sciences. It has important applied relevance in understanding and evaluating the implementation of material practices across a range of settings. | This article sets out a theory of normalization processes that proposes a working model of implementation, embedding and integration in conditions marked by complexity and emergence. The theory focuses on the *work* of embedding and of sustaining practices within interaction chains, and helps in understanding why some processes seem to lead to a practice becoming normalized while others do not. |  | Normalization Process Theory provides a robust and replicable ecological framework for analysing the dynamic collective work and relationships involved in the implementation and social shaping of practices. It is a theory for empirical application rather than abstract critique. It focuses attention on organized and organizing agency in the production and reproduction of the implementation, embedding (or not), and continuing integration of material practices. |
| 30 | Melrose et al., 2012 | Becoming Socialized into a New Professional Role: LPN to BN Student Nurses’ Experiences with Legitimation | Qualitative | This paper presents findings from a qualitative descriptive study that explored the professional socialization experiences of Licensed Practical Nurses (LPNs) who attended an online university to earn a Baccalaureate degree in nursing (BN), a prerequisite to writing the Canadian Registered Nurse (RN) qualifying exam. | The project was framed from a constructivist worldview and Haas and Shaffir’s theory of legitimation. Participants were 27 nurses in a Post-LPN to BN program who came from across Canada to complete required practicums. Data was collected from digital recordings of four focus groups held in different cities. | Transcripts were analysed for themes and confirmed with participants through member checking. Two overarching themes were identified and are presented to explain how these unique adult learners sought to legitimize their emerging identity as Registered Nurses (RNs). | First, Post-LPN to BN students need little, if any, further legitimation to affirm their identities as “nurse.” Second, practicum interactions with instructors and new clinical experiences are key socializing agents. |
| 31 | Normoyle 2009 | BECOMING A CLINICAL PSYCHOLOGIST: MIDLIFE WOMEN'SNARRATIVES OF PROFESSIONAL IDENTITY DEVELOPMENT | Qualitative | This dissertation is a narrative study of the professional developmentalexperiences of women who were becoming clinical psychologists in midlife. The role ofgender in professional identity development as described in gender socialization, thesocial construction of gender and developmental theories formed the conceptual basis forthe study. | 12 women who were between the ages of 38-53 and who werenear completion of, or had completed their doctoral training in clinical psychology were interviewed. Thesemi-structured interviews were analyzed using in-depth content and process analyses ofcore themes within cases and across cases. | The findings revealed six principle elementsof the process of professional identity development and personal change: (1) Desire andMotivation, (2) Coping and Adjustment, (3) Relationship and Connection, (4) Isolationand Difference, (5) Milestones and Transitions, and (6) Accounting and Integration. Thetextual content showed that these women encountered a complex and ongoing processthat brought change to both their personal and professional lives and identities. Thepursuit of the doctorate in midlife brought a unique set of challenges and all of theparticipants made sacrifices and adjustments within their personal life to accommodatethe demands of doctoral training. | Evidence of role conflict emerged from the narrativesas the women told of their struggle to juggle family roles with the demands of doctoral training. As graduates and early career psychologists, the participants' professional identity was just beginning to emerge. |
| 32 | O’Brien and Battista, 2020 | Situated learning theory in health professions education research: a scoping review | Review | At a time when cognitive and behavioral theories dominated the landscape of learning theories, Lave and Wenger’s situated learning theory (SLT) opened new territory by illuminating social and cultural learning processes. Many researchers and educators in the health professions embraced this theory, but few have considered *how* SLT has been used and *what* has been learned or improved through its use in health professions education (HPE). In this scoping review, the authors examine these questions and consider opportunities for future work with SLT in HPE. | The authors followed Levac’s modified scoping review framework. They conducted a search of CINAHL, ERIC, PubMed, Scopus, and Web of Science for articles referencing SLT or related concepts in HPE between 2006 and 2016. Two reviewers screened all titles and abstracts, then screened full text for inclusion based on substantive treatment of SLT. | Two authors extracted, analyzed and synthesized data from the final set of 193 articles. The authors identified two patterns of theory use. One sought an understanding of social learning processes in a specific context. The other aimed to design and/or evaluate interventions associated with communities of practice. These patterns are similar to other literatures (e.g., general education, healthcare, and organizational development) and, according to Lave and Wenger, may reflect confusion about the purpose, stance, and terminology of SLT. The authors discuss their findings in relation to primary source literature and consider opportunities for HPE researchers to employ and contribute to SLT going forward. | The authors found that most research in HPE used SLT and its related concepts (e.g., CoP, LPP, Identity) in a limited or narrow capacity, as an analytic heuristic (i.e., using the theory to clarify learning phenomena and contexts) or as a prescriptive heuristic (i.e., to guide the development and design of an educational intervention or using CoP as the intervention). While these uses are consistent with other professional domains, the authors found some conflicts with the original and continuing intentions of SLT’s progenitors when authors applied and tested CoP as a prescribed intervention. With these findings in mind, four potential future pathways for HPE stakeholders to capitalize on the conceptual affordances of SLT and CoP were suggested and they also address some of the tensions the scoping review uncovered. It is believed that a more robust application of SLT in HPE is not only possible, but holds strong potential for addressing several contemporary challenges confronting HPE. |
| 33 | Ogden et al., 2023 | Examining the nexus between medical education and complexity: a systematic review to inform practice and research | Review | Medical education is a multifarious endeavour integrating a range of pedagogies and philosophies. Complexity as a science or theory (‘complexity’) signals a move away from a reductionist paradigm to one which appreciates that interactions in multi-component systems, such as healthcare systems, can result in adaptive and emergent outcomes. This examination of the nexus between medical education and complexity theory aims to discover ways that complexity theory can inform medical education and medical education research. | A structured literature review was conducted to examine the nexus between medical education and complexity; 5 databases were searched using relevant terms. Papers were included if they engaged fully with complexity as a science or theory and were significantly focused on medical education. All types of papers were included, including conceptual papers (e.g. opinion and theoretical discussions), case studies, program evaluations and empirical research. A narrative and thematic synthesis was undertaken to create a deep understanding of the use of complexity in medical education. | Eighty-three papers were included; the majority were conceptual papers. The context and theoretical underpinnings of complexity as a relevant theory for medical education were identified. Bibliographic and temporal observations were noted regarding the entry of complexity into medical education. Complexity was relied upon as a theoretical framework for empirical studies covering a variety of elements within medical education including: knowledge and learning theories; curricular, program and faculty development; program evaluation and medical education research; assessment and admissions; professionalism and leadership; and learning for systems, about systems and in systems. | There is a call for greater use of theory by medical educators. Complexity within medical education is established, although not widespread. Individualistic cultures of medicine and comfort with reductionist epistemologies challenges its introduction. However, complexity was found to be a useful theory across a range of areas by a limited number of authors and is increasingly used by medical educators and medical education researchers. This review has further conceptualized how complexity is being used to support medical education and medical education research. This literature review can assist in understanding how complexity can be useful in medical educationalists’ practice. |
| 34 | Pittman and Foubert, 2016 | Predictors of Professional Identity Development for Student Affairs Professionals | Cross-sectional | This study examined whether professional involvement, supervision style, and mentoring predicted the professional identity of graduate students and new professionals in student affairs. | 542 questionnaire responses regarding the degree to which several variables predicted the professional identity development of student affairs professionals were gathered from graduate students and alumni. Three predictor values, being the role of mentors, supervision style, and professional involvement were considered. | Results of the study show that all three independent variables predicted the professional identity development of graduate students. | The supervision style of a supervisor, but not mentoring or professional involvement, significantly predicted the professional identity development of new professionals. Implications and recommendations for future research and student affairs practice are provided. |
| 35 | Priest and Tara, 2000 | PROFESSIONAL AND ORGANIZATIONAL SOCIALIZATION AND THE ROLE OF THE SCHOOL PSYCHOLOGIST | Qualitative | This researcher sought to explore the relationship of the professional and organizational socialization of school psychologists to their commitment to students, formal and informal activity, and the degree of role conflict they experience. This study is a replication o f a study conducted by Pincus (1994), who studied school psychologists in the New York City Public Schools. The problem posed is how do these two socialization processes of school psychologists affect their professional roles within a bureaucratic school environment that was under various constraints? | "Data were collected by means o f a modification of a survey developed by Pincus (1994) and mailed to the 694 school psychologists employed by New York’s Nassau and Suffolk County public schools." | Regression analyses revealed that professional socialization was associated with an increase in role conflict, and a decrease in support for both formal and informal activity' as well as student commitment. Organizational socialization was associated with a decrease in role conflict, support for informal activity, and commitment to students. An increase in support for formal activity was found. However, the effect of organizational socialization was twice to three times that of professional socialization. These findings support those of Pincus (1994). | The findings support the view that earlier developed norms supporting student commitment and informal activity may be weakened by professional and organizational socialization. The finding that professional socialization decreased support for formal activity supports that professional socialization may also interfere with the development of norms that support organizationally mandated tasks. The findings support those of Pincus (1994). The professional and organizational socialization of school psychologists may be socializing them away from providing important services to students and their families. If the organization does not permit school psychologists to provide a variety of services to students and if professional socialization does not support commitment to students, then students and their families may not be receiving the concern and the services they require. |
| 36 | Sadeghi et al, 2019 | Professional socialization: an analytical definition | Qualitative | Given the diversity in defining the concept of "professional socialization" in scientific literature and the use of different terms by researchers, policymakers and educational experts in the medical community, it would be useful to clarify this concept. Therefore, providing a good analytical definition for this concept through disambiguation could provide opportunities for improving educational programs and supporting strategies in this process. The aim of this study was analysing the concept of professional socialization and proposing an analytical definition for it. | Literature review of 21 articles on professional socialisation. The Walker and Avant’s eight-step concept analysis method was employed. | In this study, six conceptual areas were identified for the concept of professional socialization: identity, attributes, verbs, contents, outcomes, and affecting factors. Professional socialisation is also non-linear, cyclical, a developmental process. There is no direct relationship between chronological time and the strength of professional socialization outcomes, and the elements of the outcomes are not all of the same weight | Professional socialisation is the process through which a layperson gradually becomes a professional, and adapts to a reference point for the particular values and behaviours of the role. Clarifying this concept and its elements was the mission of this study. |
| 37 | Santivasi et al, 2022 | Reframing professional identity through navigating tensions during residency: A qualitative study | Qualitative | An individual’s identity formation has both psychological and sociological influences. Social psychology may be useful to explore the interactions between the psychological and sociological aspects of PIF. This paper investigates how resident physicians navigated tensions between professional ideals and the reality of medical practice to characterize PIF during residency training. | Using constructivist grounded theory, the authors conducted 23 semi-structured interviews with internal medicine residents. Social Cognitive Theory was utilised – specifically the bidirectional influence between person, behaviour, and context – to analyse relationships among themes. Theoretical insights were refined through group discussion and constant comparison with newly collected data. | Residents described tensions experienced during residency between pre-existing ideals of “*a good doctor*” and the realities of medical practice, often challenging residents to reframe their ideals. The authors provide evidence for the presence of dynamic, bidirectional influences between identity (person), behaviour, and environment (context), and demonstrate how PIF is informed by a complex interplay between these elements. The authors present two examples to demonstrate how residents reframed their ideals during residency training. | Residency is a critical time for the developing physician, exposing learners to multiple tensions that exist between the ideals and the realities of medical practice. Social cognitive theory provides a framework to explore PIF in residency, highlighting the bidirectional influences between identity, behaviour, and context. As residents engage in clinical experiences, receive feedback, and follow role models, they reframe their ideals while navigating tensions faced during residency. Sensitivity to these processes and assessment of the context of medical education can provide educators with opportunities to facilitate residents’ intentional reflection on professional identity and recognize and mitigate negative influences on PIF. |
| 38 | Schubert et al, 2023 | Interrogation in clinical supervision sessions: Exploring the construction ofclinical psychology trainees’ professional identities | Qualitative | Professional socialisation is a complex process through which learners become healthcare professionals.This process occurs in relational spaces: learners are exposed to norms and expectations of them ininteractions during formal and informal learning activities. Research exploring socialisation into the healthcareprofessions is extensive yet inadequately captures details of the actual processes of socialisation and forming ofprofessional identity. Our study offers a moment-by-moment examination of clinical psychology trainee supervisionto provide a deeper exploration of novice healthcare professionals’ identity development within formaleducational interactions.Aims: We sought to explore how, and in what ways, supervision interactions impact clinical psychology trainees’professional identity development. | Our data comprises 12 audio recordings of supervisor-trainee interactions in a clinical psychologytraining clinic. Initial data engagement identified 20 instances of interrogation instigated by supervisors (i.e.,repetitive questioning, enquiry) as being key events in which identities are contested and re-negotiated. Thesesections were transcribed verbatim. Drawing on positioning, framing and impression management theories, weexplored identity construction in interrogations using discourse analysis. | Supervisors approached interrogations of trainees from either a quality control or a reflective framefocusing on the client or trainee respectively. These frames influenced the positions being made available totrainees. Reflective frame interrogations often defaulted back to quality control. Trainees employed impressionmanagement tactics to manage perceived face-threat. | Findings highlight challenges with maintaining a reflective space in supervision during interrogations.Supervisors had authority to initiate interrogations that could be taken up, negotiated or resisted by trainees,ultimately maintaining certain professional knowledge and truths. We illuminate the ways in which socialisationprocesses at the interactional level during formal learning activities ultimately make available (and restrict)certain ways for trainees to think, feel and be. |
| 39 | Stubbing et al, 2018 | Authoring the identity of learner before doctor in the figured world of medical school | Qualitative | Identity formation is about how people come to make sense of themselves, how they ‘figure’ out who they are as they interact with the ‘world’(s) they are part of and with others who both exist within and outside of these worlds. Medical students are positioned between worlds, between being adolescents in the lay world of patients and becoming doctors and professionals. A consequence of this transitional position is that medical students are continually negotiating or authoring their identities in terms of who they think they are and how they portray themselves to others in relation to their training and as future doctors | The study took place at one medium-sized UK medical school offering a five-year undergraduate program (the norm in the UK). The program is systems- and case-based, with early clinical exposure to acute hospital wards and a patient home visit in the first year. In 2013, all 169 first year medical students were invited to participate in a five-year longitudinal study via a whole- class presentation, poster advert, and class email. Twenty-three students self-selected to take part in the study (14 female and 9 male participants aged between 17 and 26 years). This paper reports on the first year of this study. We used focus groups for data collection to allow participants to lead responses, discussion, and also to stimulate potentially inaccessible thoughts. Data collection was longitudinal with two sets of focus groups. | Template analysis revealed three overarching themes and associated subthemes: preconceptions of being a doctor, e.g. to help/make a difference and to be a leader. Tensions experienced with preconceptions included being unable to help/make a difference, feeling a sense of pressure to help/make a difference and lacking power. Moreover, analysis revealed participants negotiating tensions and self-authoring identities. | This paper explored participants’ internal preconceptions associated with becoming a doctor. We revealed how these preconceptions can conflict with a newly developing professional identity throughout the first year of medical school. Our findings highlight the importance of supporting students to embrace the formation of a ‘learner’ identity, a necessary part of the process of becoming a doctor. |
| 40 | Taylan and Özkan, 2022 | “I Felt Alienated in the Operating Room”: A Phenomenological Qualitative Study | Qualitative | The study was conducted to investigate the clinical practice experiences of nursing students in the operating room (OR) and to evaluate these experiences in terms of professional socialization. The OR is a complex system that requires multidisciplinary teamwork in a physical environment and coordinates the person, technology, and patient to achieve optimal outcomes. It is also a stressful learning environment for students, where mistakes are not allowed. This study aimed to investigate the clinical practice experiences of nursing students in the OR and to evaluate these experiences in terms of professional socialization. | This qualitative study was based on Heidegger's hermeneutic phenomenological approach. In the study, the Van Manen method was used to reveal how students were affected by their OR experiences. The second-year nursing students of a nursing school in the spring semester of the 2019-2020 academic year constituted the study sample. The data were collected through a semi structured interview form. Students were interviewed on the day following the clinical experience on how they perceived the OR rotation and how they were affected in the end. | This study was conducted with a total of nine students, including four males and five females, to investigate how nursing students perceived the effect of the OR clinical rotation on their professional socialization. Two themes and seven subthemes were derived in the study. The themes included (1) short and difficult experience and (2) very important for professional socialization. | It was understood that the students perceived OR practice as a short and difficult experience, felt worthless and alienated, and realized that there was no empathy in the OR. Additionally, they stated that the team members did their job very well and that they felt the importance of being the best. Moreover, they found OR practice important for guiding one's career goals, professional socialization, and gaining a professional identity. |
| 41 | Ten Cate et al., 2011 | How self-determination theory can assist our understanding of the teaching and learning processes in medical education. AMEE Guide No. 59 | Review | Self-determination Theory (SDT), designed by Edward Deci and Richard Ryan, serves among the current major motivational theories in psychology. SDT research has been conducted in many areas, among which are education and health care, but its applications in medical education are rare. The potential of SDT to help understand processes in medical education justifies this Guide. SDT is explained in seven principles, one of which is the distinction of three innate psychological needs of human beings: for competence, for autonomy and for relatedness. Further, SDT elaborates how humans tend to internalise regulation of behaviour that initially has been external, in order to develop autonomous, self-determined behaviour. | Implications of SDT for medical education are discussed with reference to preparation and selection, curriculum structure, classroom teaching, assessments and examinations, self-directed learning, clinical teaching, students as teachers and researchers, continuing professional development, faculty development and stress among trainees. |  | The lens of SDT provides a different view of processes in medical education. SDT stresses the importance of creating feelings of competence, autonomy and relatedness in medical students. Despite the large body of research in the domain of this theory, including many studies in health care, the applications seen currently in medical education are scarce. Through logical reasoning and applying the elements of the theory, the authors have proposed several hypotheses to understand processes in education that have high face validity, which are justifiable for discussion. Much of medical education methods and curricular structures applied in medical schools are constructed with a focus on practicality and based upon tradition. Some modern approaches are more theory-based, but many lack such foundation. And even if they have, there is often little evidence to predict that one method will yield superior results, compared to other methods, as medical education research is not rocket science. This should not restrain educators from seeking mechanisms to understand what could cause successes and failures in medical education. |
| 42 | Thrasher et al, 20156 | Graduate-Assistant Athletic Trainers’ Perceptions of Professional Socialization in the Collegiate Setting: Part I | Qualitative | The purpose of our study was to explore and gain an in-depth understanding of the professional socialization process of GAs in the collegiate setting (i.e., providing patient care for an intercollegiate athletic team). | Data were collected via individual semi structured phone interviews that lasted approximately 45 to 60 minutes. The semi structured interview guide was based on the research questions as well as on prior socialization research. Interviews, which were recorded and transcribed verbatim, were conducted until data saturation occurred. Data were analysed using a descriptive phenomenological method described by Giorgi. | Four themes emerged: (1) role identity, (2) initial entry into role, (3) maturation, and (4) success. | Professional socialization in athletic training depends largely on the interactions of novice ATs with experienced ATs at the organization.67 Socialization of GAs begins before graduation from their professional program, as they envision their roles and determine the expectations of their supervisors. As GAs enter the collegiate setting, organizational socialization includes formal and informal processes of orientation, athletic training experience, and didactic coursework. The GAs are able to gain confidence and feel validated in the AT role as they gain more independent clinical experience and receive positive feedback. |
| 43 | Toubassi et al, 2023 | Professional identity formation: linking meaning to well‐being | Conceptual | Trainee distress and burnout continue to be serious concerns for educational programs in medicine, prompting the implementation of numerous interventions. Although an expansive body of literature suggests that the experience of meaning at work is critical to professional wellbeing, relatively little attention has been paid to how this might be leveraged in the educational milieu. | A literature review was conducted to propose a new theoretical model. | Using the widely accepted tri-partite model of meaning, we outline how this process can contribute to wellbeing. We suggest strategies to optimize the influence of PIF on wellbeing, offering curricular suggestions, as well as ideas regarding the respective roles of communities of practice, teachers, and formative educational experiences. | Attempts to improve trainee well-being through adaptive professional identity formation (or through any other means) would be incomplete without due attention to the staggering systemic factors at play. We have proposed that professional identity formation enhances trainee experiences of meaning, and in so doing, supports their more general wellbeing. |
| 44 | Trede et al, 2012 | Professional identity development: a review of the higher education literature | Systematic review | This study examined the extant higher education literature on the development of professional identities. Higher education programmes are required to produce graduates who display mastery of theoretical ideas, competence in applying theory in complex workplace settings, and professional dispositions that foster ethical and reflective professional practices Such preparation includes learning professional roles, understanding workplace cultures, commencing the professional socialization process and educating towards citizenship. All these aspects of work preparation can be understood as contributing to the development of professional identities. | A systematic approach to identifying the literature was developed in close consultation with a university librarian. The search also held ‘higher education’ or ‘tertiary education’ or ‘university education’ as constant in order to exclude articles not concerned with university education. | Our analysis revealed that there is a dearth of journal articles in the higher education literature that comprehensively explore the development of professional identity through higher education. Rather than focusing on professional identity development, the journal articles we reviewed only tangentially discussed it by exploring dimensions such as professional reasoning, personal epistemology development, socialization into professions and learning theories. | Overall, our literature review highlights the underdevelopment of the research base for professional identity development formation. We suggest that it is likely that a host of constructive insights and messages for teaching and learning would be derived by working with a more explicit conceptualization of professional identity than has hitherto been the case in the higher education literature. |
| 45 | Wahid et al, 2017 | Professional identity formation of medical teachers in a non-Western setting | Qualitative | Understanding and supporting professional identity formation (PIF) among medical teachers has become increasingly important in faculty development programs. In this study, we explored medical teachers’ PIF in Indonesia. | We conducted a qualitative descriptive study using focus group discussions (FGDs) at four medical schools in Indonesia. Basic science and clinical teachers from four different schools were selected to participate. Data were transcribed, coded, and analysed to develop themes and subthemes. | We found that PIF among medical teachers was characterized by four factors: an internal dialogue between intrinsic values and external influences that motivates medical teachers to teach; a process of early socialization that empowers them to perform this role; experiential workplace learning that reinforces their choice; and envisioning their future to strengthen their teaching careers. | Our findings suggested that PIF among medical teachers in a non-Western setting is a continuous and dynamic process that is shaped by key socialization factors (e.g. role models, workplace learning, peer support), with significant influences from religious beliefs, family values, and societal recognition. |
| 46 | Wald 2015 | Professional Identity (Trans)Formation in Medical Education: Reflection, Relationship, Resilience | Commentary | A fundamental goal of medical education is the active, constructive, transformative process of professional identity formation (PIF). Medical educators are thus charged with designing standardized and personalized curricula for guiding, supporting, and challenging learners on the developmental professional identity pathway, including the process of socialization. | A review of existing literature was conducted to offer a commentary on professional identity transformation in medical education. | Relationships influence adoption of professional values within PIF. Key drivers of PIF include experiential and reflective processes, guided reflection, formative feedback, use of personal narratives, integral role of relationships and role models, and candid discussion within a safe community of learners (an “authentic community”). Development of PI relates to well-being (with a strong sense of shared social identity, for example, as a factor in stress buffering), and well-being relates to PIF. Reciprocal feedback loops amongst the themes of reflection, relationship, and resiliency support PIF. | It is my hope that this special issue (as a “GPS” on that journey) offers tangible insights and sparks fresh dialogues and creativity as we join with our learners (and connect with ourselves as lifelong learners) to actualize positive transformative learning environments and help crystallize and integrate affect-laden threshold moments of challenge and triumph supporting healthy PIF. |
